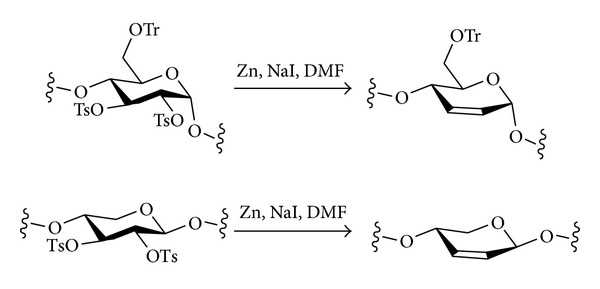# Chemical Modification of Polysaccharides

**DOI:** 10.1155/2013/417672

**Published:** 2013-09-10

**Authors:** Ian Cumpstey

**Affiliations:** Private Practice, UK

## Abstract

This review covers methods for modifying the structures of polysaccharides. The introduction of hydrophobic, acidic, basic, or other functionality into polysaccharide structures can alter the properties of materials based on these substances. The development of chemical methods to achieve this aim is an ongoing area of research that is expected to become more important as the emphasis on using renewable starting materials and sustainable processes increases in the future. The methods covered in this review include ester and ether formation using saccharide oxygen nucleophiles, including enzymatic reactions and aspects of regioselectivity; the introduction of heteroatomic nucleophiles into polysaccharide chains; the oxidation of polysaccharides, including oxidative glycol cleavage, chemical oxidation of primary alcohols to carboxylic acids, and enzymatic oxidation of primary alcohols to aldehydes; reactions of uronic-acid-based polysaccharides; nucleophilic reactions of the amines of chitosan; and the formation of unsaturated polysaccharide derivatives.

## 1. Introduction

With increasing oil prices and forecasts of a future lack of availability, renewable non-petrochemical-based alternatives to materials synthesis could become more important. Polysaccharides are the products of a natural carbon-capture process, photosynthesis, followed by further biosynthetic modifications. Some are produced on a very large scale in nature, and some have industrial relevance with, for example, materials and food applications, either in their native or chemically modified forms. This review covers methods for the chemical modification of polysaccharides. The topic of general modification of polysaccharides has been reviewed previously [1], and several more specific reviews are referenced later. In this review, I have limited myself to discussing the synthesis of modifications whereby the polymeric chain remains intact—or at least while degradation may take place to some extent, the products are still polysaccharides. The conversion of polysaccharides into small molecules has been reviewed elsewhere [2, 3] and is not covered here.

Chemical modification can change the character of the polysaccharides, for example, rendering them hydrophobic [4]. Some such processes, such as the formation of cellulose esters (including nitrocellulose, celluloid, cellulose acetate), are very well known and have been carried out at an industrial level for more than a hundred years. The object of this review is not to cover such well-known processes in detail but rather to describe published results of current research and the state-of-the-art in polysaccharide derivatisation. Neither have I gone into details about the possible applications of the products but have focussed on aspects related to reactivity and chemical structure. The modifications are presented classified by reaction type.

**Figure 1:**
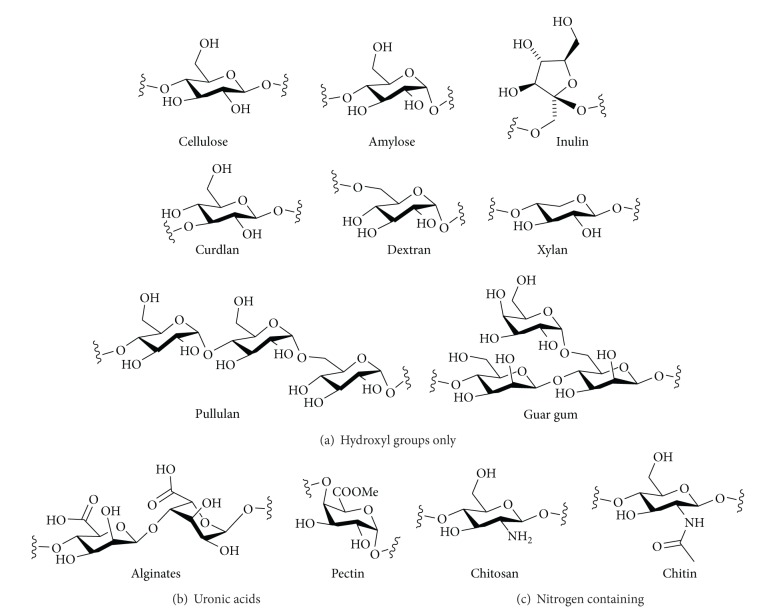
Structures of the repeating units of some of the polysaccharides discussed in this review. Some of the structures are simplified (see text): branching is not shown for dextran, xylan, and pectin; the alginate structure shown shows the two linkage types rather than a formal repeating unit; the chitin and chitosan structures shown represent extremes of a continuum of structures.

The structures of the natural (native) polysaccharides whose chemical modification is described in this review are shown in Figure 1. Structurally, the simplest molecules consist of a monosaccharide repeating unit with hydroxyl groups as the only functional groups. Examples of this type of structure include the very common polysaccharides, cellulose and amylose. Other related structures such as curdlan, a (*β*1–3)-linked glucan, and inulin, the only polysaccharide based on furanosides considered here, also have simple structures. Amylopectin has a structure similar to amylose (these to polysaccharides are the components of starch), but it is a branched structure, with many amylose-like chains linked together by (*α*1–6)-branching points. Dextran is basically an (*α*1–6)-linked glucan, but it may have a little or a lot of branching at the secondary hydroxyl groups. For example, in so-called regular comb dextran, every residue in the backbone is substituted by an (*α*1–3)-linked glucose unit. Xylans, a component of hemicelluloses, are made up of a (*β*1–4)-linked xylopyranose backbone, but it also may be branched, for example, by 4-*O*-methylglucuronic acid, or acetylated to a greater or lesser degree. Xylose is a pentose, so the pyranose units in xylan do not have a primary hydroxyl group. Guar gum and locust bean gum both consist of (*β*1–4)-linked mannan backbones substituted by Gal(*α*1–6) units to some extent. In guar gum, approximately every other mannose residue is substituted with galactose, whereas in locust bean gum, long unsubstituted regions alternate with regions of heavy galactose branching.

Pullulan, alternan, and lichenan are glucans with more than one type of glycosidic linkage in the polysaccharide backbone. Pullulan is an unbranched polysaccharide with three monosaccharides in the repeating unit, [Glc(*α*1–4)Glc(*α*1–4)Glc(*α*1–6)]. Alternan has two monosaccharides in its repeating unit, [Glc(*α*1–3)Glc(*α*1–6)], but it can also contain some Glc(*α*1–3) branching. Lichenan is an unbranched polysaccharide based on glucose with mixed (*β*1–3) and (*β*1–4) linkages.

Some polysaccharides have other functional groups as well as the simple hydroxyl groups. Alginates and pectins are based on uronic acids; their monosaccharide constituents are all oxidised at C-6 to the carboxylic acid level. Alginates consist of domains of (*α*1–4)-linked l-guluronic acid interspersed with domains of (*β*1–4)-linked mannuronic acid. Pectins are polysaccharides rich in galacturonic acid, although this acid commonly will be found as its methyl ester. A simple backbone of (*α*1–4)-linked galacturonic acid methyl ester may also be substituted by other monosaccharide branches.

A very common polysaccharide based on aminosugars is chitin/chitosan. The chitin/chitosan relationship can be regarded as a continuum, with polysaccharides containing more of the free base being called chitosan and those mostly *N*-acetylated being called chitin.

The extent of derivatisation reactions is given in terms of the degree of substitution (DS). The DS is defined as the number of substitutions made per monomer unit. The maximum DS will depend on the structure and reaction in question. For example, cellulose has three hydroxyl groups per monomer, so in an acetylation of cellulose, all three may be acetylated, and the maximum DS would be 3. But only one of the alcohols is primary, so in an oxidation reaction that only acted on primary alcohols, the maximum DS would be 1. The degree of polymerisation (DP) is another important factor, giving an average length (expressed in number of monomer units) of the polysaccharide. A loss in DP during a reaction indicates that degradation of the polysaccharide backbone has occurred. It has been pointed out that many reports use cellulose with low DP (which is more soluble) and also that many do not comment on whether there is any decrease in DP during derivatisation reactions [5]. Order of magnitude values of the DP of cellulose samples could be *ca* 280 for Avicel and *ca* 2000 for cotton linters [6].

If uniform partial derivatisation is to be achieved, it can be important that the reaction is conducted in homogeneous solution. This is less important if the goal is complete derivatisation of a polysaccharide. Running a reaction under homogeneous conditions may also lead to a cleaner product, due to fewer side reactions, as less forcing conditions (lower reaction temperature and lower excesses of reagents) may be necessary than in heterogeneous reactions; hence, the choice of an appropriate solvent for a polysaccharide substrate is important. “Swelling” of solid material by a solvent will not dissolve the solid to give a homogeneous solution, but it will nevertheless increase the accessibility of the reactive groups of the polymer to reagents in solution.

Polysaccharides are often insoluble in water or organic solvents, so solvent mixtures can be used. Non-aqueous solvent mixtures that dissolve cellulose often consist of an organic liquid and an inorganic salt. Examples include DMA (dimethylacetamide)/LiCl; DMF/LiCl; DMI (1,3-dimethyl-2-imidazolinone)/LiCl; and DMSO/TBAF (tetrabutylammonium fluoride). The DMSO/Et_3_N/SO_2_ mixture is a salt-free solvent for cellulose. It will sometimes be necessary to heat to high temperature (150°C) before cellulose will dissolve in these solvents, but it will then remain in solution on cooling. Inorganic salts are often formed as by-products in derivatisation reactions (e.g., stoichiometric NaCl would be formed in a benzylation reaction using NaOH/BnCl), so when they are added at the start of a reaction to aid solubility, this is unlikely to cause problems in itself.

Similar solvents or solvent mixtures to those used for cellulose are often used for other neutral polysaccharides, but many are more soluble and some are water-soluble. Starch is generally more soluble than cellulose. Solvents for chitin include LiCl (5%)/DMA, LiCl/*N*-methyl-2-pyrrolidone, CaCl_2_/MeOH, and hexafluoroisopropyl alcohol.

Charged polysaccharides such as chitosan (which may be protonated on nitrogen) or polyuronates such as alginates (which can form carboxylate salts) will have very different solubility properties. Hence, chitosan is soluble in aqueous organic or mineral acids below pH 6.5 and also in DMSO.

Ionic liquids (room-temperature ionic liquids) are relatively new solvents that have found use in polysaccharide chemistry [7, 8]. They can dissolve polysaccharides, including cellulose, hemicellulose, and wood, allowing derivatisation reactions to take place under homogeneous conditions. Cellulose dissolves in ionic liquids, aided by conventional heating, microwave irradiation, or sonication, with up to 25% (w/w) being obtained in [bmim]Cl [9]. Other ionic liquids gave 5–10% w/w solutions of cellulose. The properties of ionic liquids can be fine-tuned by structural modification of one or other of the two ionic components. Increasing the length of the alkyl chains in the cation component resulted in a less efficient dissolution of cellulose. Amylose was shown to have a very high solubility in ether-derived ionic liquids. Ionic liquids have been called “green” solvents due to their recyclability and low vapour pressures (low volatility), but a low vapour pressure can limit the recyclability of a solvent, as it can make its purification difficult after it has been used in chemical reactions. As a result, volatile and distillable ionic liquids have been designed for polysaccharide derivatisation [10].

## 2. Saccharide Oxygen as Nucleophile

This section covers the formation of ethers and esters, in which the saccharide oxygen acts as a nucleophile in the reaction and is retained in the product. Different degrees of reaction can be considered. As well as very low DS, where only a few hydroxyl groups per polysaccharide chain are derivatised, and maximum DS, where all the hydroxyl groups are derivatised, and points in between these extremes, we can consider regioselective reactions in which a single hydroxyl group on each monosaccharide residue reacts preferentially to (say) near completion. Regioselective reactions allow the synthesis of structurally well-defined products. But more than this, if a regioselective reaction goes to completion, reaching DS = 1, then it can be followed in principle by further derivatisations that do not have to be selective, but that can nevertheless introduce further functionality at specific positions in a polysaccharide structure.

There is a significant disadvantage of working with polysaccharides when it comes to matters of regioselectivity. In monomeric molecules, when a reaction gives incomplete regioselectivity (resulting in the formation of regioisomers, disubstituted or trisubstituted products, etc.) the desired product may be purified from the other components of the product mixture by crystallisation or chromatography. In polysaccharides, any such purification is impossible, as correctly modified monosaccharide residues of the polysaccharide will be covalently linked to incorrectly modified monosaccharide residues. This means that only the most regioselective modifying reactions may be used for polysaccharide modification if a homogeneous polysaccharide structure is required.

### 2.1. Etherification

**Figure 2:**
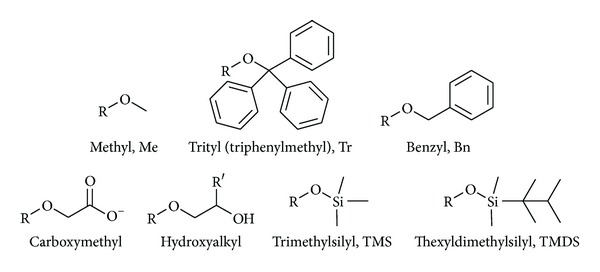
Structures of some of the ethers discussed in this review.

Etherification involves the reaction of an alcohol (here a saccharide alcohol) with an alkylating agent in the presence of a base ((1); Figure 2)



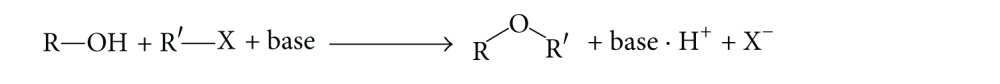
(1)


Typical alkylating agents include alkyl halides (chlorides, bromides, iodides) or, less commonly, alkyl sulfonates. Normally, a strong base will be used to deprotonate the alcohol to give the alkoxide. Alkylation reactions generally have a poor water-compatibility, as water can hydrolyse the alkylating agent.

#### 2.1.1. Alkyl and Benzyl Ethers

The formation of cellulose ethers under homogeneous conditions in typical nonderivatising solvents is possible, but it is more problematic than ester formation (see below). The solvent of choice for cellulose etherification appears to be DMI (1,3-dimethyl-2-imidazolidinone)/LiCl [11]. In this solvent, much lower excesses of reagent were required than with alternative solvents. First the cellulose was dissolved by briefly heating to 150°C. Treatment with NaOH and MeI for 5 h at 70°C gave 2,3,6-tri-*O*-methylcellulose with a DS of 3. It should be pointed out that when the NaOH was added, the cellulose crashed out of solution to some extent, and so the reaction was in fact not entirely homogeneous.

Complete etherification (i.e., tri-*O*-alkylation) of cellulose was also investigated in other solvents for etherification with various alkyl groups. Different solvents and bases were evaluated in the benzylation reaction, and the best conditions of those tested were found to be powdered NaOH and BnCl (both in an excess of 10 equiv./hydroxyl), in a solvent of DMSO/SO_2_/Et_2_NH, heating at *ca* 80°C for 3-4 h [12]. DMSO/N_2_O_4_ and DMA/LiCl gave slightly worse results. Subsequent papers covered the formation of substituted benzyl ethers and allyl ethers [13] and of simple alkyl ethers [14] of cellulose, all under essentially the same reaction conditions. Purification was achieved by extraction into chloroform, precipitation after the addition of EtOH, and then washing with water, EtOH and hexane.

In DMA/LiCl, methyl, hydroxyethyl, and hydroxypropyl ethers of cellulose could be formed under homogeneous conditions, using iodomethane or the epoxides as alkylating agents [15]. But high excesses of reagents were required, slow reactions were seen, and only products with low DS values (1.1–1.7) were accessible. A DMSO/LiCl solvent was used for the homogeneous etherification (methyl, ethyl, propyl, and butyl peretherification) of cellulose, using dimsyl sodium (from NaH and DMSO) as base [16].

Ionic liquids have been tested as solvents for the etherification of polysaccharides (cellulose and starch) under basic conditions but with little success to date, in contrast to esterification reactions (see below) [17].

Other polysaccharides have also been shown to undergo peretherification reactions under similar conditions. Xylan was benzylated using BnBr, NaOH, and 18-crown-6 in DMSO [18], and amylose was converted into its tri-*O*-benzyl derivative by treatment with NaOH and BnCl in DMSO [19]. A detailed investigation into the benzylation of starch in water (NaOH, BnCl) was reported [20]. As expected, widespread hydrolysis of the BnCl occurred under these conditions.

The benzylation of chitin was reported [21]. *β*-Chitin was suspended in DMSO, and sodium hydride (5 equiv.) and benzyl chloride (10 equiv.) were added. After heating at 60°C for 24 h, the product (DS = 1.33) was obtained by precipitation from MeOH. When more NaH (7 equiv.) was used, a product with DS = 2 was obtained, but *N*-alkylation is likely to occur as well as *O*-alkylation under such reaction conditions. Alternatively, chitin was suspended in DMSO and treated with KOH; this insoluble deprotonated chitin was then filtered and washed to remove water, then it was resuspended in DMSO and BnCl was added. This method gave the product with a DS of up to 0.8 [22].

Considering other alkyl ethers, amylose and starch were treated with propyl bromide and NaOH in DMSO to give propyl ethers with DS of up to 3.0 [23]. The purification of polysaccharides with high DS was achieved by precipitation from water, but those with low DS were more difficult to purify. Pullulan was converted into its propyl and butyl ethers with DS between 1 and 2.6 by treatment with the alkyl bromides and NaOH in H_2_O/DMSO [24].

#### 2.1.2. Carboxymethyl Ethers

Carboxymethyl cellulose is an industrially important ionic cellulose ether, and the synthesis of this type of derivative based on some hemicellulose polysaccharides has been investigated to some extent. The synthesis of carboxymethyl ethers of xylan was investigated under homogeneous conditions (in water) or slurry conditions (in *i*-PrOH or EtOH/toluene) using NaOH as base and ClCH_2_COONa as alkylating agent [25]. Guar gum was derivatised with carboxymethyl ethers in water or in EtOH/toluene (as for xylan, above) to give a product with a DS of 0.8. Repeating the procedure gave further substitution and a product with a higher DS [18]. Konjac glucomannan was derivatised with carboxymethyl ethers in methanol to give a product with a DS of 0.3 [18].

#### 2.1.3. Hydroxyethyl Ethers

Other than cellulose derivatives, which are produced industrially by epoxide-ring opening, guar gum and xylan were etherified (up to DS = 2) by treatment with ethylene oxide or propylene oxide and sodium hydroxide [18].

### 2.2. Esterification

**Figure 3:**
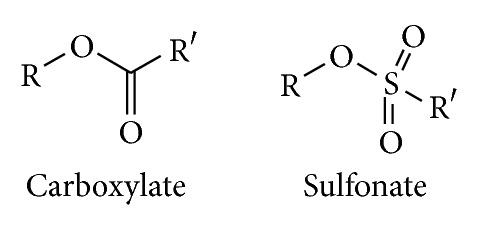
General structures carboxylate and sulfonate esters.

Esterification in general will involve the reaction of an alcohol (here a saccharide alcohol) with an acylating agent ((2), Figure 3)



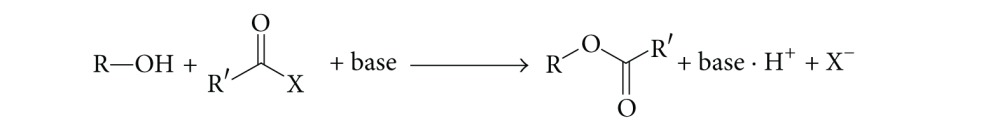
(2)


#### 2.2.1. Acetate and Other Carboxylate Esters

Carboxylate esters can be formed using carboxylic acids as acylating agents under strong-acid catalysis (Fischer esterification) or by using an activated derivative such as an acid chloride or anhydride, either with base or with a Lewis acid.

The strong-acid catalysis method is used to produce cellulose acetate, an important industrial product [26]. But this method does not produce the triacetate, due to partial transient sulfation during the reaction. Cellulose triacetate can be prepared in a similar way using an acid catalyst that does not covalently attach to the cellulose, such as HClO_4_.

When an activated carboxylic acid derivative (e.g., acid anhydride, acid chloride) reacts with an alcohol under basic conditions, the base should be present in a stoichiometric amount (it will be protonated by the acid by-product of the reaction), but it can be a weak base, such as pyridine or triethylamine.

(*1) Homogeneous Reactions*. Cellulose carboxylates (DS of up to 2.4–2.8) were prepared by the reaction of cellulose under homogeneous conditions in DMA/LiCl solution with acid chlorides and triethylamine or with acid anhydrides and sulfuric acid [27]. The cellulose carboxylate products were purified by precipitation into water followed by Soxhlet extraction into methanol. Similarly, starch was esterified with acyl chlorides and pyridine in DMA/LiCl solution at 100°C for 6 h, followed by purification by precipitation [28]. With long-chain fatty acid chlorides, DS values of up to 3 were seen.

Xylan acetates with DS of up to 2 (i.e., complete acetylation) could be prepared either with Ac_2_O/pyridine in DMF/LiCl or under acid catalysis in AcOH [29]. Alternatively, a xylan acetate with high DS (*≈*1.9) and clean ^1^H NMR spectra was prepared using Ac_2_O and pyridine in DMF [30]. With longer-chain acyl chlorides, xylan reacted under homogeneous conditions in DMF/LiCl to give polysaccharides with lower DS values (0.3–1.5) [31].

Vinyl carboxylates have also been used as acyl donors, reacting spontaneously with cellulose in DMSO/TBAF to give polysaccharides with DS values of up to 2.6 [32].

The acetylation of cellulose in an ionic liquid solvent, [amim]Cl (1-allyl-3-methylimidazolium chloride), was achieved in 2004 using acetic anhydride to give products with DS of *ca* 2.5–2.7 [33]. The esterification of cellulose in ionic liquids is straightforward for short-chain esters [34]. Several ionic liquids gave similarly good results, with [bmim]Cl (1-butyl-3-methylimidazolium chloride) being the best. Acetic anhydride or acetyl chloride reacted with cellulose without any added base within 2 h at 80°C to give cellulose acetates with DS of up to 3. However, only lower DS values (e.g., 1.6 for lauryl chloride) were obtainable with fatty acid chlorides in ionic liquids, presumably because the partially acylated polysaccharide becomes more and more nonpolar until it precipitates out of the polar ionic solvent, stopping the reaction.

The use of carboxylic acids themselves as acylating agents rather than derivatives such as acid anhydrides or acyl chlorides could be attractive, as the acids may have a wider availability and be more soluble in polar solvents. The Fischer esterification using the carboxylic acid as solvent and with strong-acid catalysis has already been mentioned, but *in situ* activation of carboxylic acids under mild conditions can also be used for polysaccharide acylation. When tosyl chloride was used as an activating agent for with various long-chain carboxylic acids in a DMSO/TBAF solvent, acylated celluloses with DS of up to 2.6–2.9 could be formed [32, 35]. Cellulose reacted with carboxylic acids using classic peptide coupling reagent, DCC, in nonaqueous solvents (e.g., DMA/LiCl) to give derivatised polysaccharides with low DS values. Starch was acylated under similar conditions by the *in situ* activation of carboxylic acids with TsCl or CDI (carbonyldiimidazole) [28].

The acetylation of alginates was less straightforward than for neutral polysaccharides [36]. The solubility of alginates can be changed by changing the ionisation state (i.e., acid versus salt) and (for the salt form) the counterion [e.g., sodium versus tetrabutylammonium (TBA)]. TBA-alginates were soluble in DMSO/TBAF, but DMA/LiCl did not dissolve either the acid or salt (Na or TBA) forms. When the alginate solution was treated with Ac_2_O and pyridine, only low DS of up to *ca* 1 were obtained. It is worth mentioning here that DMSO can react with acylating agents to generate a Swern-type oxidant that can destructively oxidise polysaccharide hydroxyl groups.

In a method for the selective *O*-acylation of chitosan [37], the polysaccharide was suspended in water, and a carboxylic acid (C_2_–C_9_ as well as some halogenated or unsaturated acids) and H_2_SO_4_ (2 M) were added at room temperature. The mixture was then stirred at 80°C for 4 h, and the products (with low DS values of 0.02–0.2) were purified by pH adjustment, precipitation from acetone, and Soxhlet extraction. Under these conditions, the nucleophilicity of the nitrogen is blocked by protonation.

(*2) Heterogeneous Reactions*. In a heterogeneous reaction, the starting polysaccharide is insoluble in the reaction solvent. But then dissolution may or may not occur during the course of the reaction; only surface groups may be acylated, or alternatively bulk hydroxyls may also react (due to solvent swelling of the material); the macroscopic structure of the material may be retained after derivatisation (fibre, paper, cloth, or nanofibrils, etc.).

Heating a suspension of insoluble cellulose in a mixture of pyridine and acylating agent (5 equiv./Glc = 1.3 equiv./OH) can give acylated celluloses with some acylating agents after purification by precipitation from water [38]. Polysaccharides with DS values of 2.6–2.9 were obtained with acetyl chloride and with long-chain acyl chlorides (>C_10_) after 3 h. With pivaloyl chloride, a much longer reaction time was required to obtain a product with DS = 2.5 in low yield, and with shorter chain acyl chorides (<C_6_), decomposition was seen. A similar synthesis of cellulose esters was reported from a suspension of the polysaccharide in pyridine and the acid chloride [39, 40], while initially heterogeneous cellulose reacted with acetyl chloride without added base to give cellulose acetates with DS values of up to 2.96 [32].

Konjac glucomannan was acylated with palmitoyl chloride and pyridine in benzene in a heterogeneous reaction in which the polysaccharide dissolved during the course of the reaction to give a product with DS up to 2.7 [18]. Arabinoxylan was fully esterified under Fischer conditions by suspending the polysaccharide in a carboxylic acid anhydride (acetic, propionic, butyric) and treating with catalytic methanesulfonic acid [41]. Also here, the polysaccharide dissolved during the course of the reaction. Mixed anhydrides generated from a carboxylic acid and other more reactive acids (e.g., trifluoroacetyl) have also been used as acylating agents with polysaccharides under heterogeneous conditions [42].

#### 2.2.2. Sulfonate Esters

Sulfonate esters can act as leaving groups in S_N_2 reactions (see below), and many of their applications derive from this aspect of their reactivity. They may be introduced with reasonably good regioselectivity for the primary hydroxyl groups, and regioselective sulfonate syntheses are described in the section on regioselective reactions (see below). But polysaccharide sulfonates with DS >2 are also accessible. The most commonly seen sulfonates in polysaccharides are toluenesulfonates (tosylates, Ts) and methanesulfonates (mesylates, Ms) [43].

The classic reaction conditions for tosylate formation involve heating the (initially heterogeneous) polysaccharide with tosyl chloride in pyridine. Three possible side reactions that may occur during sulfonate ester formation, all arising from nucleophilic displacement of the formed sulfonate ester are as follows: (i) cyclisation by attack of one of the secondary hydroxyl groups (e.g., O-3); (ii) attack by pyridine to form a C-6 pyridinium salt; (iii) attack by chloride to form a C-6 chloride. These side reactions are a result of the long reaction times and high temperatures required for the heterogeneous reaction.

Thus, these side reactions can be minimised or suppressed by using homogeneous conditions [44]. Tosylation and mesylation reactions of cellulose in solution in DMA/LiCl gave uniform and well-defined products with DS values between 0.4 and 2.3. The tosylation of cellulose underhomogeneous conditions in the ionic liquid [amim]Cl was also recently achieved [45].

Sulfonate esters of other polysaccharides have also been synthesised. Chitin was tosylated under homogeneous conditions in DMA/LiCl [46], dextran tosylates were prepared in organic solvent without any added salt [47], and konjac glucomannan was tosylated to give products with DS of up to 2.3 [18]. The mesylation of cross-linked particles of pullulan has been reported [48].

### 2.3. Regioselective Etherification and Esterification of Polysaccharides

The primary alcohol of a saccharide will, with very few exceptions, always be more nucleophilic than the secondary alcohols. The difference in reactivity between the primary and secondary alcohols can vary, though, and complete regioselective distinction between primary and secondary alcohols (i.e., normally C-6 *versus* all of C-2, C-3, and C-4) will often not be seen. The respective rate constants for the substitution of primary and secondary alcohols do not change during a reaction, and if a primary alcohol reacts more quickly than a secondary alcohol, its concentration will decrease more rapidly as the reaction progresses. Therefore, as the reaction progresses, the rates of reaction of the primary and secondary alcohols will become similar and regioselectivity will decrease.

Differentiation between the nucleophilicity of the different secondary hydroxyl groups in a polysaccharide will often be difficult or impossible, and polysaccharides containing different substitution patterns may often be formed. Having said that, there are a few examples of regioselectivity between the secondary positions of polysaccharides that can be exploited synthetically. The regioselective protection of cellulose, focussing on ether and ester protecting groups has been reviewed [45] and covered to some extent in other reviews [49, 50].

The hydroxyl groups of cellulose are much more reactive in solution than they are in the solid phase, because when cellulose dissolves, the extensive hydrogen-bonding network is broken up. As a result, reactions in solution can be carried out under milder conditions than in the solid phase, and this allows a higher degree of selectivity. Thus, regioselective derivatisations of cellulose and other polysaccharides are generally carried out under homogeneous reaction conditions, and the solvent system DMA/LiBr (or LiCl) is often used.

Only a rather limited number of groups/transformations live up to the very high regioselectivity criteria that are necessary for the modification of polysaccharides. These include the installation of trityl ethers (at O-6) and of bulky silyl ethers (at O-6 or at both O-2 and O-6). The installation of carboxylate esters (at O-6, but not normally selective enough) and tosylate esters (at O-6, but not normally completely selective, or at O-2) are also considered here. The installation of halides at C-6 in a phosphane-mediated reaction is also often a regioselective process, but this in this reaction, the polysaccharide behaves as an electrophile, so it is considered in a later section of this review.

#### 2.3.1. Trityl Ethers

**Scheme 1:**
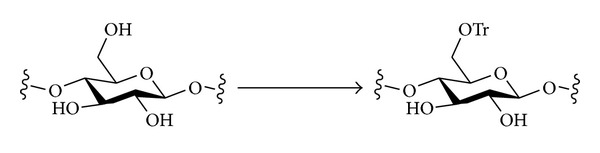


The trityl group reacts with cellulose preferentially at the primary hydroxyl, O-6, on steric grounds (Scheme 1). Trityl ethers may be installed by heating cellulose (rayon) with pyridine and trityl chloride (i.e., under initially heterogeneous conditions with dissolution occurring as the reaction proceeds), and DS values close to 1 with little substitution of the secondary positions are obtainable [26, 51]. Cellulose has also been tritylated under homogeneous conditions to give products with DS values of 1.0 [6]. The solvents used were DMSO/N_2_O_4_; DMA/LiCl; or DMSO/SO_2_/DEA.

6-*O*-Trityl derivatives of some other polysaccharides have been prepared, directly or indirectly. Amylose underwent tritylation regioselectively at O-6 uneventfully [52]. Chitin was also tritylated regioselectivity with reaction at O-6 [21]. *β*-Chitin was suspended in pyridine and heated at 90°C for 72 h with trityl chloride (10 equiv.) and DMAP (3–6 equiv.). Products with DS values of 0.75–1.0 were obtained by purification by precipitation from methanol. A 6-*O*-trityl derivative of chitosan was prepared by a three-step sequence. First, the nitrogen was protected as a phthalimide derivative, then O-6 was tritylated, and finally *N*-deprotection gave the 6-*O*-tritylchitosan with DS = 1 [53].

#### 2.3.2. Silyl Ethers

**Scheme 2:**
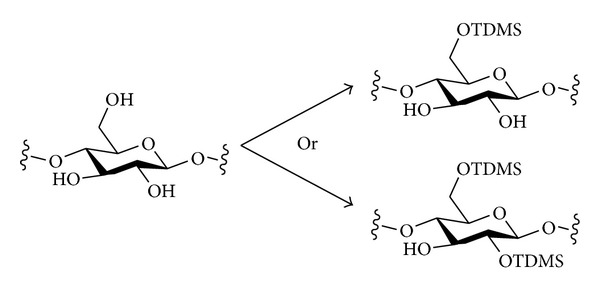


Thexyldimethylsilyl chloride (TMDSCl) has been shown to react with cellulose with very good regioselectivity, and different regioselectivities, O-6 only or for both O-2 and O-6, are seen under different reaction conditions (Scheme 2). Treatment of cellulose (undissolved, i.e., under initially heterogeneous conditions) with TMDSC1 in DMF saturated with ammonia at –15°C resulted in the introduction of TMDS groups at C-6 only, with a DS of 0.99 [54]. When the reaction was carried out under homogeneous conditions in DMA/LiCl and with imidazole as base, 2,6-di-*O*-thexyldimethylsilylcellulose was formed with a DS of 2.0 [55, 56]. Moreover, this 2,6-protected derivative can be used for the regiospecific introduction of substituents at O-3 of cellulose. 3-*O*-Methylcellulose and 3-*O*-allylcellulose have been synthesised in this way. The silyl ethers can be removed by treatment with TBAF (tetrabutylammonium fluoride). Liquid ammonia has also been used as an effective solvent for silylation reactions of cellulose [57].

#### 2.3.3. Carboxylate Esters

Regioselectivities (for O-6) are generally lower for carboxylate esterification reactions of cellulose than those seen for the formation of trityl ethers or silyl ethers [45]. An investigation of various sterically hindered acylating agents, including pivaloyl chloride, adamantoyl chloride, and 2,4,6-trimethylbenzyl chloride, in solvents including DMA/LiCl, DMSO/TBAF, and the ionic liquid [amim]Cl, failed to give satisfactory regioselectivity [58]. But having said that, in a different study, excellent regioselectivity for O-6 of cellulose was observed in an esterification reaction using benzyl chloride in [amim]Cl without any added base [59].

It is relevant in this context to note that silyl ether protection may be regiospecifically replaced by carboxylate protection [60]. When a cellulose derivative bearing trimethylsilyl ethers is treated with an acyl chloride in the absence of a base, the silyl ethers are regiospecifically replaced by acyl groups (in the presence of a base, the silyl ethers remain, and the free hydroxyl groups are acylated). But while this process is well known for trimethylsilyl ethers, it has apparently [45] not yet been investigated for thexyldimethylsilylethers, which (as described above) can be introduced into cellulose with excellent regioselectivity.

#### 2.3.4. Sulfonate Esters

Cellulose reacts preferentially at O-6 in tosylation reactions (see above), but the regioselectivity is not perfect. The esterification of cellulose with various sulfonic acid chlorides, including the 2,4,6-trimethylbenzenesulfonyl group, under homogeneous conditions (in DMA/LiCl), was investigated in an attempt to improve the regioselectivity for substitution at O-6 [61], but in general, the products contained mixtures of 2- and 6-tosylation.

**Scheme 3:**
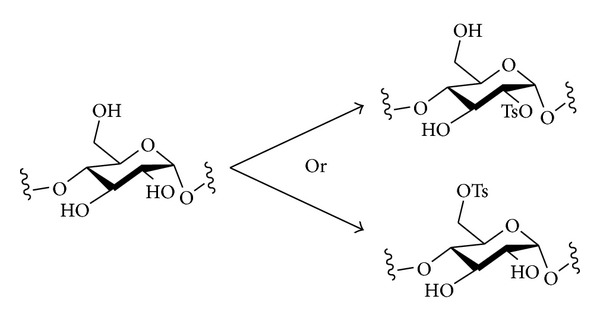


A very interesting result has been obtained concerning the regioselectivity of the tosylation of starch. When starch (70% amylose) was tosylated in solution in DMA/LiCl, O-2 reacted preferentially, with very good regioselectivity (over O-3 and O-6), to give a product with a DS *≈* 1, with the tosyl groups essentially exclusively at C-2. The regioselectivity was proved by ^1^H and ^13^C NMR spectroscopy (Scheme 3) [62]. This regioselectivity is counterintuitive, and apparently, it is also solvent-dependent: Horton had previously reported that when the tosylation of amylose was carried out in pyridine, the more expected product, 6-*O*-tosyl-amylose, was formed with DS *≈* 0.6 (Scheme 3) [63].

Inulin was tosylated by treatment with TsCl and Et_3_N, in DMF/LiCl at 0°C. Purification by precipitation then dialysis gave a polysaccharide product derivatised at O-6 and with some partial derivatisation at O-4 [64].

### 2.4. Enzymatic Reactions: Regioselective Esterification and Deesterification

In general, regioselectivity in chemical reactions is controlled by a combination of steric, electronic, and stereoelectronic factors. In enzymatic reactions in contrast, the reaction will occur at the position that is held close to the relevant catalytic amino acid side chains when the substrate is bound in the active site of the enzyme. That is true at least when the substrate of the reaction is the same as, or close in structure to, the natural structure that the enzyme has evolved to modify, for example, for galactose-6-oxidase and galactose (see below). Some enzymes, though, have broad substrate tolerance and catalyse reactions on rather generic structures. When esterases, lipases, and proteases are used to catalyse the formation and hydrolysis of esters on polysaccharides in the laboratory, this is not the natural function of the enzyme, so they have not evolved to differentiate the different hydroxyl groups. Rather, in these enzyme-catalysed reactions, the enzyme will tend to act on the hydroxyl group (for esterification) or ester (for hydrolysis) that is most sterically accessible, i.e., those at the primary positions. Hence in principle, 6-monoesters may be accessible by enzyme-catalysed regioselective acylation of an unprotected polysaccharide, and in principle, 6-mono-unprotected polysaccharides may be accessible by peracylation followed by regioselective hydrolysis of the primary esters.

The considerations regarding solvents for enzyme-catalysed reactions can be summarised briefly as follows. Enzymes normally require at least a trace of water to function properly, and they may also be structurally unstable in nonaqueous media. However, water is not a good solvent for acylation reactions, as the enzyme-catalysed reactions are reversible. When water is present in excess (i.e., as solvent), the equilibrium would lie towards hydrolysis, so the DS values of the products would be very low. Polar solvents (e.g., DMF, DMSO, etc.) can strip the essential catalytic water from the surface of enzymes, rendering them inactive. Solvents with lower hydrogen-bonding ability will thus be more likely to lead to higher enzyme activity, but those with a better hydrogen-bonding ability would better dissolve the polysaccharide substrates. Thus, in choosing a solvent, a balance must be struck between dissolving the substrate and maintaining the activity of the enzyme [4, 65].

Nonpolar solvents are not ideal, as the enzyme and the substrate are insoluble, and insoluble enzymes cannot catalyse reactions on insoluble substrates. But enzymes can be made soluble in nonpolar solvents by micelle formation, or they can be made accessible by immobilisation in the pores of a solid surface (as in Novozyme, i.e., immobilised *Candida antarctica* lipase B).

#### 2.4.1. In Nonpolar Solvents

In a pioneering approach to the enzymatic modification of solvent-insoluble polysaccharides in organic solvents [66], a method was developed to use surfactants to solubilise enzymes in organic solvents. In this way, insoluble amylose could be acylated with a protease from *Bacillus subtilis* (Subtilisin Carlsberg) using vinyl caprate as acyl donor in isooctane as solvent. As the starting polysaccharide is completely insoluble in the very nonpolar solvent, only surface-accessible hydroxyls could be acylated, and the authors estimated that >90% of the surface-accessible primary hydroxyls were esterified. This corresponded to DS values of *ca* 0.15 and 0.30, respectively, for a thin amylose film and a milled amylose powder. Subsequently, the enzymatic esterification of various solid celluloses was addressed, including cloth, thread, paper, and milled particles [67]. The cellulose samples failed to react in isooctane, but esterification did occur in pyridine when the Subtilisin Carlsberg (protease) was transferred into that more polar solvent, presumably due to better preswelling of the cellulose.

In a related approach, the enzymatic acylation of starch in toluene was achieved by coating polysaccharide nanoparticles in surfactant [68]. “Reverse-micelles” were formed with the starch particles and the surfactant in octane, and then the octane was removed. These surfactant-coated particles then underwent acylation in toluene at 60°C with immobilised *C. antarctica* lipase B (i.e., Novozyme 435), using vinyl esters or acid anhydrides as acyl donors. A DS of up to 0.9 was obtained with acylation occurring regioselectively at O-6. Nanoparticles have a high surface area/volume ratio, which allows efficient derivatisation of a heterogeneous system.

#### 2.4.2. In Water

Enzyme-catalysed esterification reactions are reversible, so in water, the DS values of the products will tend to be very low. The esterification of starch in water using decanoic acid as acyl donor, catalysed by a lipase from *Thermomyces lanuginosus*, was reported. Only very low DS (=0.018) was obtained [69]. The authors compared different methods of measuring the DS, including the classic titrimetric method (saponification followed by back titration) and NMR and FT-IR based methods, and proposed a new method based on ester hydrolysis followed by GC analysis. The acetylation of (insoluble) cellulose in water using vinyl acetate as the acyl donor, catalysed by a lipase from *Aspergillus niger,* was reported. But here again, only very low DS values were seen (quoted as 0.16% by weight) [70, 71].

#### 2.4.3. In Polar Aprotic Solvents

A series of papers describe the lipase-catalysed esterification of starch with fatty acids, either in polar aprotic solvents (DMSO or DMF) or under solvent-free conditions with microwave heating. The esterification of starch was investigated using lipases from *Thermomyces lanuginosus *[72], *Burkholderia cepacia *[73], and *Candida rugosa *[74]. Carboxylic acids obtained by the hydrolysis of coconut oil were used as acyl donors. Both neat (DS = 1.0–1.5) and solution (DS = 1.0–1.45) methods resulted in significant esterifcation of the starch, except for when *T. lanuginosus* was used in solution in DMSO, when only a low DS (0.08) was obtained.

The free hydroxyl groups of cellulose acetate were acylated using Novozyme (immobilised *Candida anctarctica* lipase B) in acetonitrile [75].

#### 2.4.4. In Ionic Liquids

Ionic liquids might seem to be a promising candidate for this transformation, as they can dissolve polysaccharides, and they are good solvents for the regioselective enzymatic acylation of unprotected monosaccharides. When conventional organic solvents are used for the enzymatic acylation of unprotected monosaccharides, the initial reaction products (typically 6-*O*-acyl derivatives) will tend to be more soluble than the starting material in the reaction solvent and so are more available for further reaction. This can result in overacylation (to give, e.g., 3,6-di-*O*-acyl derivatives) and mixtures of products. But ionic liquids dissolve the starting monosaccharides, so the reaction mixtures are homogeneous and good regioselectivity results [9]. However, the regioselective enzymatic acylation of polysaccharides in ionic liquids does not appear to have been investigated.

#### 2.4.5. Enzymatic Deesterification of Polysaccharides

An example of the cleavage of esters from 6-*O*-acyl-cellulose (i.e., only O-6 acylated) using a protease is reported in the literature [67]. Partial hydrolysis occurred in water, and the authors concluded that the more accessible surface esters were cleaved from the heterogeneous (insoluble solid) substrate.

A very interesting development concerns esterases that have naturally evolved to hydrolyse the esters of polysaccharides. Xylan in hemicellulose can be partially substituted by glucuronic acid residues and by acetates. Acetyl xylan esterases are enzymes that hydrolyse these acetates at the 2- and 3-positions of xylopyranose in xylan. Several of these enzymes were screened for cleavage activity of ester groups in partially acetylated celluloses (DS = 0.7 or 1.4), and some of the enzymes showed regioselective behaviour, as shown by ^13^C NMR spectroscopy [76]. The xylan esterase from *Aspergillus oryzae* cleanly cleaved the O-2 and O-3 acetates, leaving the O-6 acetate. Other xylan esterases (e.g., from* Schizophyllum commune *or* Aspergillus niger*) cleaved the O-2 acetate, leaving the O-3 and O-6 acetates (albeit less cleanly).

## 3. Saccharide Carbon as Electrophile

The replacement of a saccharide oxygen by a heteroatomic nucleophile in a nucleophilic substitution (S_N_) reaction typically requires at least two steps. First, a saccharide hydroxyl group must be transformed into a good leaving group, which results in the attached carbon becoming susceptible to nucleophilic attack. Second, treatment with a nucleophile results in attack at the electrophilic carbon of the polysaccharide and displacement of the leaving group.

Saccharide electrophiles are much less reactive towards nucleophilic displacement than their more typical hydrocarbon-derived counterparts. In considering the reactions of polysaccharides, we consider nucleophilic substitution reactions at the primary and secondary positions (but not the anomeric position) of the constituent monosaccharides. In contrast to typical hydrocarbon substrates, saccharides will almost certainly never undergo nucleophilic substitution by an S_N_1 mechanism at the secondary positions nor at the primary positions. This is because an intermediate carbocation would be strongly destabilised by the multiple electron-withdrawing hydroxyl groups. Hence all nucleophilic substitution at the primary and secondary positions in a polysaccharide will occur by S_N_2 processes.

Even S_N_2 reactions are disfavoured in saccharides, at the primary positions, and very much so at the secondary positions. The empirical effect, sometimes called the *β*-oxygen effect or Oldham and Rutherford's rule [43, 77, 78], has electronic and steric explanations, which I summarise very briefly here. In an S_N_2 reaction, electrons must be relocalised onto the departing leaving group, and this aspect of the mechanism is disfavoured by having electron-withdrawing groups in the vicinal positions [79]. Also, the bulk of neighbouring alkoxy or acyloxy groups makes saccharide-derived electrophiles less reactive in S_N_2 reactions (*cf* the neopentyl effect in S_N_2 reactions of hydrocarbons). A further factor that disfavours S_N_2 reactions at the secondary positions of pyranoses (but not furanoses) derives from the well-known high stability of a six-membered ring in the chair conformation, especially one bearing multiple equatorial substituents. At the S_N_2 transition state, a ring-conformational change occurs to accommodate the nucleophile and leaving group in the coordination sphere of the central carbon. This ring-conformational change is less favourable in a six-membered ring due to the loss in the stability of the molecule in moving away from a very stable to a less stable ring-conformation.

Thus, S_N_2 reactions at the secondary positions of polysaccharides are almost unknown, but the fact that they can be achieved in high yields in monosaccharide systems, using good nucleophiles and good leaving groups, means that this could be a possible avenue for future exploration in the synthesis of polysaccharide derivatives. The derivatisation of cellulose by nucleophilic substitution (saccharide electrophile) has been reviewed [80].

### 3.1. Installation of Leaving Groups

Leaving groups that are useful at the primary positions include bromide, iodide, less reactive sulfonates, or phosphonium leaving groups generated *in situ* (in Mitsunobu and related reactions). Leaving groups that are useful at the secondary positions of monosaccharides are triflates and epoxides, but nucleophilic displacement at the secondary positions has hardly been exploited in the polysaccharide series, with only a rare example of a well-defined epoxide-opening reaction by an oxygen nucleophile (see below). Thus, almost all of the nucleophilic substitution chemistry of polysaccharide electrophiles that has been reported to date has taken place at the primary positions.

#### 3.1.1. Sulfonates

Hydroxyl groups react with sulfonating agents to generate sulfonate esters. It may be possible to activate the primary alcohol (OH-6) regioselectively, but for more details on this process, see the section above on nucleophilic reactions of polysaccharide hydroxyl groups. The sulfonate group has a general structure RS(O)_2_O–, and the R group can be varied to tune the electronic properties and thus the reactivity of the sulfonate ester. Despite the almost unlimited possibilities for structural variation here, only a few sulfonates have been in common usage in the nucleophilic displacement reactions of polysaccharides.

Mesylate (methanesulfonate, R = Me) and tosylate (*p*-tolunesulfonate, R = *p*-MeC_6_H_4_) have broadly similar reactivities and will normally undergo nucleophilic displacement at the primary positions but not at the secondary positions of pyranosides. When there are free hydroxyl groups at the vicinal positions to tosylates or mesylates at the secondary positions of partially protected monosaccharides or polysaccharides, nucleophilic substitution may take place. Presumably, though, this process goes *via* epoxide intermediates, as when there is no vicinal alcohol group, there is no substitution reaction. Triflate (trifluoromethanesulfonate, R = CF_3_) has a strongly electron-withdrawing R group. Consequently, it is a better leaving group, and it can be used in nucleophilic substitution reactions at the secondary positions of monosaccharides, but examples on polysaccharide substrates do not appear to be known.

#### 3.1.2. Halides

**Scheme 4:**
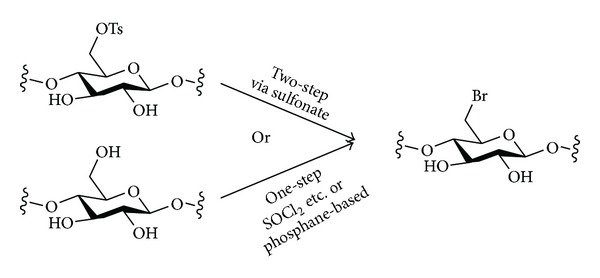
Introduction of halides, illustrated for the bromination of cellulose.

Halides are the classic leaving groups in nucleophilic substitution reactions, and the displacement of halides from the primary positions (e.g., C-6 of cellulose, amylose, etc.) of polysaccharides has been used to introduce nucleophilic groups (Scheme 4).

One method that has been used for the introduction of the halide leaving groups at C-6 of polysaccharides is the treatment of C-6 sulfonates (including tosylates and mesylates) with halide salts using acetone as solvent (i.e., Finkelstein conditions) [43]. An obvious disadvantage of this approach, though, is that if the halide is to be used as a leaving group in a nucleophilic substitution reaction, it can seem pointless to add an extra step to a reaction sequence when the C-6 sulfonate in the starting material can itself act as a leaving group in substitution reactions with the same nucleophiles.

Thus, methods for the preparation of polysaccharide halides directly in one step from the native polysaccharides would appear to be advantageous.

In the monosaccharide series, several sets of mild reaction conditions based on treatment with PPh_3_ together with a halide source that can be reduced (e.g., CBr_4_ in the Appel reaction, I_2_ in the Garegg reaction, etc.) have been developed for the regioselective preparation of bromides or iodides from the unprotected glycosides. Under these mild reaction conditions, the primary alcohol reacts regioselectively, and the secondary alcohols remain untouched [81].

Polysaccharides may also be halogenated directly and regioselectively under related phosphane-based conditions, or using classical halogenating agents such as SOCl_2_, without initial protecting-group manipulations. In cellulose, C-6 is halogenated first, and C-3 may also be halogenated under certain conditions, while C-2 does not normally react [80]. In chitin, C-6 may be halogenated, while C-3 does not react. Thus, chitin may be transformed into a polysaccharide containing three different functional groups, halogen, alcohol, and amide, in a single step.

Cellulose could be chlorinated with the classical chlorinating agents, thionyl chloride and mesyl chloride (MsCl), to give polysaccharides with DS values of up to 2.8, meaning that almost complete chlorination had occurred at both primary and secondary positions [80]. However, significant depolymerisation was also observed under these conditions. The reagent system of *N*-chlorosuccinimide (NCS)/PPh_3_/LiCl in DMA was more regioselective for the chlorination of cellulose. 

Several other polysaccharides were chlorinated with good regioselectivity for the primary positions using MsCl, including amylose (in DMF/LiCl) [82], inulin (in DMF, 70°C, 16 h) [64], and pullulan (in DMF) [83].

The chlorination of chitin using sulfuryl chloride was investigated [84]. With this reagent, reaction at C-6 was seen at low temperatures, and at higher temperatures, C-3 was also chlorinated. Chitin could be chlorinated regioselectively at C-6 using NCS/PPh_3_ in DMA/LiCl to give a product with a DS of 1.0, but some depolymerisation was seen under these conditions [85].

The bromination of cellulose could be carried out with the tribromoimidazole/PPh_3_/imidazole reagent system in DMA/LiBr to give bromocelluloses with DS values of up to 1.6 [86]. Here, bromination had occurred at C-6 and C-3, and the brominated C-3 carbons were found to have a mixture of *gluco* and *allo* configurations.

An essentially completely regioselective bromination of cellulose (at C-6) was achieved using *N*-bromosuccinimide (NBS)/PPh_3_ in DMA/LiBr, giving a 6-bromo-6-deoxycellulose with DS = 0.9 [87, 88]. The regioselectivity of this bromination reaction can be better than that of a tosylation reaction. This makes phosphane-mediated bromination an attractive method for the very regioselective modification of cellulose (at C-6) [45]; the analogous direct iodination of unprotected polysaccharides does not appear to be known, however.

Similar bromination reactions of other polysaccharides with the NBS/PPh_3_ reagent system gave similarly excellent regioselectivity and high degrees of substitution. When amylose was treated with NBS/PPh_3_ in DMF, only derivatisation of the primary positions was observed [82], and it was possible to monitor the progress of this reaction by following the development of the NMR spectra. The analogous bromination of chitin was achieved with NBS/PPh_3_ in DMA/LiBr to give a product with a DS of 0.94, but here some loss in DP was seen [89]. It is possibly relevant that while chitin is soluble in DMA/LiCl, it is not soluble in DMA/LiBr, so this reaction was heterogeneous.

The bromination of curdlan was achieved with a different phosphane-based reagent system, CBr_4_/PPh_3_ in DMF/LiCl [90]. The reaction proceeded essentially to completion and with complete selectivity for the primary position (C-6) [90].

#### 3.1.3. Epoxides

**Scheme 5:**
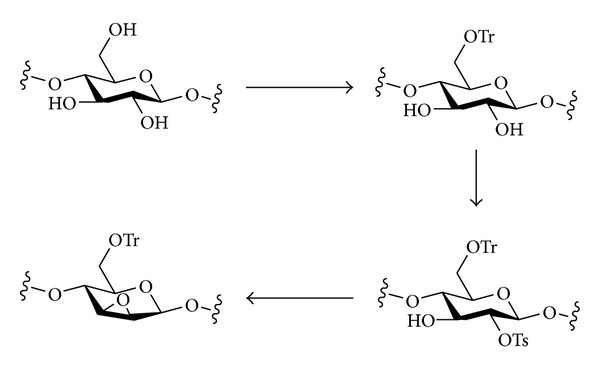
Synthesis of a cellulose epoxide (DS 0.3).

To date, polysaccharide epoxides do not appear to have been widely investigated, but the synthesis of a 2,3-anhydro derivative of cellulose (i.e., a 2,3-epoxide) has been reported (Scheme 5) [91]. First, O-6 was protected as a trityl ether, then O-2 was converted regioselectively into a tosylate. Treatment of this compound with base resulted in attack of O-3 onto C-2, displacement of the tosylate, and closure of the epoxide ring to give a 2,3-anhydro-6-*O*-tritylcellulose. The DS of this polysaccharide was *ca* 0.3, as determined from the incorporation of methyl groups after ring-opening by methoxide.

Cyclodextrin (per) epoxides are also known [92], and they have been synthesised by a similar but possibly more regioselective sequence of 6-*O*-silylation, 2-*O*-sulfonation, and base treatment for epoxide closure.

### 3.2. Nucleophilic Displacement

#### 3.2.1. Oxygen Nucleophiles

Normally, esters or ethers of polysaccharides (or indeed of monosaccharides) would be prepared by the reaction of a saccharide oxygen nucleophile with an alkylating agent or acylating agent (see above). The complementary approach, where the saccharide acts as an electrophile and is attacked by an alcohol (for ether formation) or a carboxylate (for ester formation), is much less common, but examples of this type of derivatisation do exist for polysaccharide substrates.

A situation where the more usual approach of nucleophilic attack by a saccharide oxygen nucleophile would be impossible would be in the synthesis of phenyl ethers. And indeed, a 6-*O*-phenyl ether derivative of cellulose was synthesised by displacement of a 6-tosylate by phenoxide [93, 94]. Nucleophilic substitution reactions at the secondary positions of polysaccharides are extremely rare, but a 2,3-epoxide derivative of cellulose underwent ring-opening by methoxide in a reaction that was assumed to be quantitative [91].

Intramolecular *O*-nucleophilic displacement to give cyclic derivatives is also known. For example, starch was converted into a 3,6-anhydro derivative with a DS of 0.85 using the following sequence: tritylation of O-6, acetylation of O-2 and O-3, detritylation of O-6, tosylation of O-6, and finally deacetylation of O-2 and O-3, which also resulted in intramolecular nucleophilic attack of O-3 onto C-6, displacing the tosylate and cyclisation to form the 3,6-anhydrosugar [95].

Esterification by this concept has also been reported. Carboxylate salts have been used in nucleophilic displacement reactions with primary amylose halides to give C-6 esters [82].

Finally, esterification is possible under the conditions of the Mitsunobu reaction, an overall formal condensation reaction between an (unactivated) alcohol and a carboxylic acid nucleophile. The basis of the Mitsunobu reaction is a redox reaction between stoichiometric amounts of an oxidising agent [normally DEAD (diethyl azodicarboxylate; which is reduced to DEAD-H_2_)] and a reducing agent [normally PPh_3_ (which is oxidised to Ph_3_P=O)] that require a mole equivalent of water to allow their reaction. Hence, anhydrous conditions are a prerequisite for this chemistry. The mechanism involves the *in situ* activation of an alcohol by the generation of a phosphonium leaving group and its subsequent displacement by a nucleophile to give the product. The reaction is related to the phosphane-based halogenation reactions described above.

Mitsunobu reactions at the primary positions of carbohydrates are well known. The reactions are normally high-yielding and regioselective, so it is often possible to refunctionalise the primary position of an unprotected monosaccharide [96]. However, a limited number of reactions of secondary carbohydrate alcohols are known.

Very little has been published on the Mitsunobu chemistry of polysaccharides. However, the reactivity of amylose under the conditions of Mitsunobu esterification has been investigated [82]. Initially esterification occurred regioselectively at C-6, but as the reaction proceeded above DS = 0.5, some esterification of the secondary positions started to be observed.

#### 3.2.2. Nitrogen Nucleophiles

**Scheme 6:**
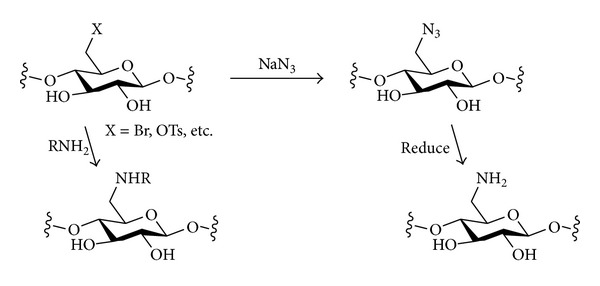
Introduction of nitrogen as alkylamines or azide.

The introduction of different types of nitrogen-containing groups at the primary positions of polysaccharides by nucleophilic displacement has been fairly extensively investigated. Two broad classes of nucleophile can be considered (Scheme 6). Amines will be neutral nucleophiles and will carry one or more alkyl chains that will be retained in the final product. Alternatively, a negatively charged nucleophile such as azide could be used. The azide in the polysaccharide product could then be reduced to reveal an amine that could be further functionalised if desired. The monovalent nature of an azide nucleophile can have the advantage of avoiding possible multiple substitution of amine nucleophiles that would lead to cross-linking and complex product mixtures [97].

A synthesis of 6-amino-6-deoxycellulose (DS = 1.0) by the essentially uniform introduction of nitrogen at C-6 of cellulose has been described [98]. Tosylation of cellulose resulted in complete derivatisation of O-6, but the reaction was not completely regioselective, and significant tosylation of O-2 and O-3 also occurred. This polysaccharide was then treated with azide. The C-6 tosylates were substituted, but the secondary tosylates did not react. Treatment with LiAlH_4_ reduced the C-6 azides to give C-6 amines and, at the same time, reductively cleaved the 2- and 3-tosylates to give the final product. The displacement of the C-6 tosylate by azide was carried out in DMSO at 50°C. When higher temperatures (100°C) or an acetone/water solvent were used, some introduction of azide at C-2 or C-3 was also seen (possibly *via* epoxide intermediates, see above).

An alternative approach to 6-amino-6-deoxycellulose going *via* the C-6 bromide, which can be formed from cellulose more regioselectively than the C-6 tosylate, has been published [99]. Thus, bromination of cellulose followed by azide displacement and reduction gave 6-amino-6-deoxycellulose with very clean ^13^C NMR spectra (DS = 0.96) in only three steps. However, some depolymerisation occurred (the Avicel microcrystalline cellulose starting material had DP = 114; product DP = 66). But when microwave irradiation was used for heating, the reaction times could be shortened, and the degradation minimised (starting DP = 114; product DP = 106) [100].

Primary halides or tosylates of several other polysaccharides have been shown to undergo nucleophilic displacement by azide. 6-Azido-6-deoxyamylose was prepared from the corresponding amylose bromide (sodium azide, DMSO, 50°C, 6 h) or chloride (sodium azide, DMSO, 70°C, 65 h [82]). As expected, the bromide was much more reactive than the chloride. Similarly, a starch tosylate reacted with sodium azide (DMF, 100°C, 24 h) to give a starch azide with a DS of 0.96 [101].

Treatment of 6-bromo-6-deoxycurdlan (DS *≈* 1) with azide gave complete substitution, as judged by the very clean ^13^C NMR spectrum of the product [90]. The introduction of azide into phthalimide-protected chitosan was achieved by displacement of both tosylate and bromide leaving groups [102]. The reaction of tosylates of lichenan, pullulan, and dextran with an azide nucleophile was investigated [61]. Heating with sodium azide in DMF (24 h, 100°C) resulted in higher degrees of substitution of tosylate by azide (67–75%) for the tosylates of lichenan and pullulan than for the tosylate of dextran (45%), probably because the number of primary tosylates in dextran [predominantly a (1–6)-linked polymer] is lower. Azide substitution of a 6-chloro-6-deoxypullulan (NaN_3_, water, 100°C) [83] and of tosyl or chloride derivatives of inulin (NaN_3_ in DMSO) [64] has also been reported.

**Scheme 7:**
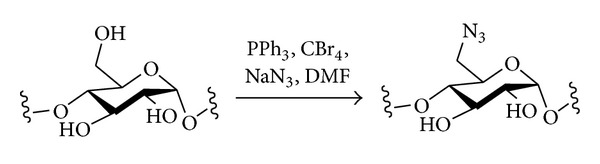
Azide formation from the hydroxyl group under Appel-like conditions.

The direct introduction of azide into unprotected polysaccharides in a phosphane-based process related to the Appel, Garegg, and Mitsunobu reactions discussed above offers an advantageous straightforward one-step route to 6-azido-6-deoxy derivatives of some polysaccharides (Scheme 7) [103]. Amylose or pullulan could be treated with PPh_3_/CBr_4_ in DMF/LiN_3_ under homogeneous conditions at room temperature to readily give the C-6 azides regioselectively. This procedure was extended to starches, replacing LiN_3_ by the more easily available NaN_3_, and using either DMF or DMA as solvent [104]. Native starches failed to react unless their granular structures were disrupted, in which case full conversion was seen. With amylose or amylopectin starches, when NaN_3_ (2 equiv.) was used and the reaction was run at 100°C for 1 h, essentially homogeneous incorporation of azide at C-6 (DS = 1) was observed; no evidence of substitution at C-2 or C-3 could be seen.

The functionalisation of cellulose derivatives (but not other polysaccharides) using amine nucleophiles has also been investigated. The reaction of tosylated cellulose with methylamine was studied in detail [typical conditions: DMA, MeNH_2_ (aq., *ca* 40 equiv.), 60°C, 48 h; purification by precipitation] [105]. The nucleophilic substitution reaction occurred only at C-6, and conditions were found that allowed the preparation of a polysaccharide with DS_N_ of *ca* 1, but presumably some unreacted tosylate groups remained at the secondary positions of this product.

Similarly, the reaction of tosylated cellulose (DS_total_ *≈* 2; DS_C-6_ = 1.0) with butylamine was studied under different conditions [106]. The reaction proceeded much more quickly (and regioselectively for C-6) in neat butylamine (neat BuNH_2_, 50°C, 24 h) than it did in DMSO solution (DMSO, BuNH_2_ (*ca* 5 equiv.), 75°C, 24 h). It has also been shown that bromide can be an effective leaving group in such reactions, as 6-bromo-6-deoxycellulose (DS = 0.92) reacts with amines in DMSO at 90°C to give, after purification by precipitation and dialysis, polysaccharide amine products with DS_N_ *≈* 0.9 [107]. Finally, tertiary amines have been shown to react with tosylated cellulose to give ammonium salts [108].

#### 3.2.3. Sulfur Nucleophiles

**Scheme 8:**
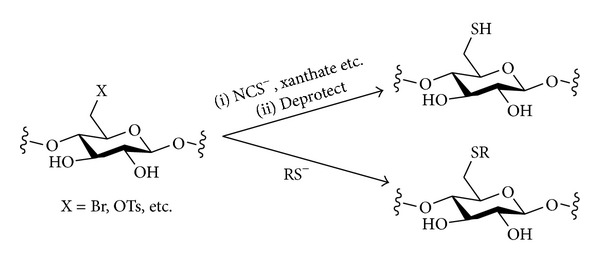
Introduction of sulfur with thiolate or other sulfur nucleophiles.

The introduction of sulfur nucleophiles into polysaccharides (cellulose and starch) has been the subject of some research (Scheme 8), albeit to a much lesser extent than for nitrogen nucleophiles.

Thiols were used as nucleophiles in nucleophilic substitution reactions with 6-bromo-6-deoxycellulose (RSH; R = Me, Ph, CH_2_CH_2_OH, CH_2_CH_2_NH_2_, etc.) under heterogeneous conditions in aqueous sodium hydroxide, giving a maximum conversion of 65% [109]. When the pH was too basic, 5,6-elimination and 3,6-cyclisation competed with the S_N_ reaction. A similar reaction between a 6-bromo-6-deoxycellulose and thiols was also carried out under homogeneous conditions in DMA/LiBr, using triethylamine as base, followed by purification by precipitation or dialysis [110]. A detailed optimisation of the conditions for this reaction was undertaken. 6-*O*-Tosyl-cellulose has also been used as an electrophile in a thioether-forming reaction with sodium methanethiolate (DMF, 0°C, 8 h) [111].

Other sulfur nucleophiles have been used in reactions with polysaccharide electrophiles for the indirect synthesis of polysaccharide thiols. 6-Bromo-6-deoxycellulose (DS = 0.85) was converted into the thiol in a two-step process. First, sulfur was introduced using a thiourea nucleophile (DMSO, 70°C, 48 h) [112]. The initial product, a (poly)thiouronium salt, then underwent hydrolysis to give the polysaccharide thiol. Alternatively, 6-bromo-6-deoxycellulose (DS = 0.92) underwent substitution with potassium thiocyanate (DMF, 150°C, 2 h) [113]. Purification by precipitation and dialysis gave a product with DS_SCN_ = 0.88 and residual DS_Br_ = 0.02. A 6-deoxy-6-thio derivative of amylose with DS = 0.8 could be prepared similarly. Thus, 6-*O*-tosyl-amylose (or alternatively 2,3-di-*O*-phenylcarbamoyl-6-*O*-tosyl-amylose) underwent a nucleophilic substitution reaction with KSCN, and then the thiocyanate product was reduced (and the 2,3-protection cleaved) by treatment with LiAlH_4_ [114]. Xanthates were used as nucelophiles in reactions with tosylates of starch (DS < 0.2), and the products were reduced to give the polysaccharide thiols [115]. Here, though, the conversion of the tosylates in the nucleophilic substitution reaction was not complete, and some formation of thioether linkages was observed.

A heterogeneous reaction in which sulfur nucleophiles were bonded to Whatman filter paper was carried out by initial chlorination followed by nucleophilic substitution by treatment with thiourea or cysteine in suspension in a DMF/water mixture [116].

It is perhaps worth noting that in monosaccharides, the introduction of thiol nucleophiles at the secondary positions of pyranosides by triflate displacement is relatively trivial [117], but related work has not been done to date in polysaccharides. Also in monosaccharides, selenoethers have been introduced in a protecting-group-minimised approach, similar to those described here for thioethers [118]. But again, no related work with selenium nucleophiles appears to have been done to date in the polysaccharide series.

## 4. Oxidation

**Scheme 9:**
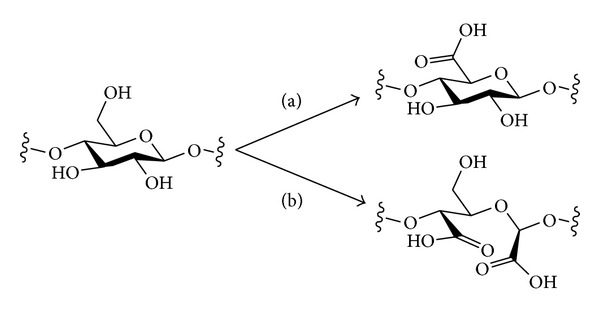
Different modes of chemical oxidation, illustrated for cellulose. (a) Oxidation of a primary alcohol; (b) oxidative cleavage of a diol.

Polysaccharides may be oxidised in different ways to produce structures of different types (Scheme 9). Where there is a free primary alcohol (e.g., at C-6 in cellulose or amylose), this may be oxidised simply to give the aldehyde or further to the carboxylic acid level. Oxidation to the carboxylic acid level would result in a polysaccharide based on uronic acids, which would then resemble the structure of natural polyuronic acids such as pectin or alginates. Chemical and enzymatic methods have both been used for oxidation of the primary alcohols of polysaccharides. An alternative mode of oxidation would be the oxidative cleavage of 1,2-diols. Where this structural motif occurs in a polysaccharide (e.g., at C-2 and C-3 in cellulose, amylose, or xylose) it may be possible to undergo a ring-opening oxidative C–C bond cleavage to give dicarbonyl compounds. With these different possible oxidation modes come issues of selectivity—when carrying out an oxidative derivatisation of a native (unprotected) polysaccharide, it would be desirable to have either one of these oxidation modes operating but not both. When oxidising primary alcohols, it may also be desirable to avoid potential simple oxidation of unprotected secondary alcohols to give ketones and also to be able to choose conditions that result in either oxidation to the aldehyde or the carboxylic acid levels. The periodate oxidation of polysaccharides [119] and the oxidation of cellulose have recently been reviewed [120].

### 4.1. Oxidation of Primary Alcohols

A method that has been used for the oxidation of C-6 of monosaccharide glycosides to the uronic acid level is treatment with oxygen over a heterogeneous platinum metal surface as a catalyst [121]. In many respects, this is an attractive method, since molecular oxygen is used as the oxidising agent, water is the sole by-product, and in principle, heterogeneous catalysts can be easily recovered and reused. However, this method has a significant disadvantage when it comes to the oxidation of polysaccharides: as the catalyst is heterogeneous, the degree of oxidation (DS_ox_) can be quite low [122]. It is generally true that homogeneous catalysts will give better results for the modification of insoluble polymeric substrates. Nevertheless, inulin with a DP of *ca* 30 could be oxidised to the uronic acid level at the primary positions (C-6) with a DS_ox_ of *ca* 0.20 under such conditions [123], and C-6 oxidation of a galactan over platinum to the uronic acid level with a DS_ox_ of *ca* 0.15 has also been achieved [121, 122, 124]. Purification was carried out by precipitation followed by membrane filtration.

In the early 1990s, Van Bekkum found that a homogeneous catalyst, TEMPO [i.e., (2,2,6,6-tetramethyl-piperidin-1-yl) oxyl], could be used for the regioselective oxidation of the primary alcohols in polysaccharides (starch and inulin were included in the initial report) to give the corresponding polyuronic acids with essentially complete conversion, (*i.e.*, DS_ox_ *ca* 1.0) [125].

In a typical oxidation procedure [126], the polysaccharide (20 mmol Glc units), a catalytic amount of TEMPO (0.65 mol-%), and NaBr (0.4 equiv.) were dissolved in water. A pH-adjusted solution of the stoichiometric oxidant, NaOCl (1.1 equiv.), was added at 0°C. The reaction mixture was kept at 0°C, and the pH was kept at *ca* 10 by the addition of NaOH. The reaction was complete after 1-2 h, after which EtOH was added to quench the reaction and to precipitate the polysaccharide, which could then be collected by filtration. The products were the sodium salts of the polyuronic acids.

The method is catalytic in TEMPO and is selective for primary alcohols, with secondary alcohols remaining unaffected. In the reaction mechanism, the persistent radical TEMPO is initially oxidised to give the active oxidant, an oxoammonium species.

This species then oxidises the polysaccharide primary alcohol to the aldehyde, being itself reduced to the hydroxylamine. The polysaccharide aldehyde must then be hydrated, and the hydrate is then oxidised to the acid by a second molecule of the oxoammonium reagent. The stoichiometric oxidant is NaOCl/NaBr, NaOBr, or NaOCl, and this is responsible for the initial oxidative activation of TEMPO and the subsequent reoxidation of the hydroxylamine to the active oxoammonium species.

This method was suitable for the very selective oxidation of C-6 of soluble potato starch and of pullulan [126]. The three Glc(A) environments of the oxidised pullulan can be clearly seen and distinguished in the ^13^C NMR spectra of the product. The selectivity for the primary alcohols was estimated to be >95%.

Amylodextrin, which is a short amylose structure with a DP of *ca* 20, was oxidised selectively at C-6, but some overoxidation at the reducing ends became significant at the shorter polymer chain length [125]. Dextran, which is basically a (1–6)-linked polymer without free primary hydroxyl groups except for end-groups, was oxidised only at the level of background oxidation of the secondary alcohols by NaOCl (or NaOBr), which occurred much more slowly than the TEMPO-catalysed oxidation of the primary alcohols [125]. Apparently, the selectivity for the primary alcohols was less good in inulin, based on furanoside residues, but when the reaction was quenched after 20 min, the a ^13^C NMR spectrum of the product was clean [125] and >90% selectivity was reported.

Water-soluble polysaccharides were investigated initially, but this C-6 oxidation to the carboxylic acid (carboxylate) level greatly increased the water solubility of the polysaccharide products, and in fact, the method was found to be broadly applicable. The polysaccharides that have been successfully oxidised using the TEMPO method include starch [125–127], amylose [127], amylopectin [127], amylodextrin [125], dextran [125], regular comb dextran [127], pullulan [126, 127], alternan [127], inulin [125], chitin [127–129], chitosan [127, 128], and cellulose [127, 128]. Normally, excellent selectivity for oxidation of the primary alcohol was seen, and normally DS_ox_ values close to 1.0 (i.e., complete conversion) were obtained [127]. Some reports indicate that the selectivity for the primary alcohols was lower in chitin, and some oxidation of secondary alcohols also occurred [127], while others found that chitin could be oxidised to give a polyuronic acid with a quite clean ^13^C NMR spectrum [128].

The oxidation of cellulose by the TEMPO method has been studied in detail [128]. Different celluloses were investigated, including microcrystalline cellulose (DP = 200), linters (DP = 800), bleached kraft pulps (DP = 900–1200), and amorphous regenerated celluloses. The oxidation procedure was essentially identical to that described above, except that all of the celluloses were, of course, initially insoluble in the aqueous reaction medium. When the oxidation was complete, the polysaccharide had dissolved and purification could be carried out again by precipitation from EtOH. The regenerated celluloses were completely oxidised at C-6 within 2 h, whereas the native celluloses did not form homogeneous solutions, even after long reaction times, presumably due to the crystallinity and the resulting inaccessibility of some of the C-6 hydroxyl groups. When the native cellulose samples had been mercerised, they underwent rapid oxidation. An essentially completely regioselective (C-6) oxidation of these insoluble polysaccharides (i.e., the regenerated or mercerised cellulose samples) was achieved under these conditions, as shown by the ^13^C NMR spectra of the products. 

It was found that under these reaction conditions, some depolymerisation occurred, presumably by a E1_CB_ elimination mechanism across C-4–C-5; the reaction time, temperature, and amounts of reagents are all important factors to be considered if this depolymerisation is to be minimized [128].

A variant of the TEMPO oxidation method in which the sodium bromide is omitted, but still using NaOCl as the stoichiometric oxidant, has been used for the oxidation of potato starch [130]. This variant method gave similar reaction rates and selectivities when the reaction was carried out at room temperature, and when the pH was kept below 9.5.

TEMPO is a persistent stable radical to the extent that it is a commercially available solid. Related methods for the oxidation of polysaccharides using shorter-lived N–O radicals have been investigated briefly. An example of such a method uses catalytic *N*-hydroxysuccinimide, NaOCl as stoichiometric oxidant, and NaBr [120]. Another related reaction is the oxidation with N_2_O_4_ [122, 124, 131, 132]. This reagent oxidises the primary position of carbohydrates regioselectively to give the uronic acids, but the regioselectivity is not perfect, and some oxidation of the secondary positions can take place. Normally then, it is necessary to include a borohydride reduction step after the oxidation to reduce any ketones back to the alcohol level (clearly this would introduce issues of diastereoselectivity and inhomogeneity in the products). Depolymerisation can also occur (by E1_CB_ elimination resulting in chain cleavage at C-4, see above) under the basic conditions of this reaction. The side-reactions that are found with this reagent mean that it is less suitable for the preparation of pure polyglucuronic acid polysaccharides than the other methods discussed here.

### 4.2. Enzymatic Oxidation

The enzyme galactose-6-oxidase (EC 1.1.3.9) catalyses the C-6 oxidation of galactose to the *aldehyde* level, using oxygen as the oxidant and generating hydrogen peroxide as the reduced by-product (3). The reactions are carried out in aqueous solution. Thus, the reaction is complementary to the TEMPO oxidation, where the product of C-6 oxidation is the carboxylic acid rather than the aldehyde.



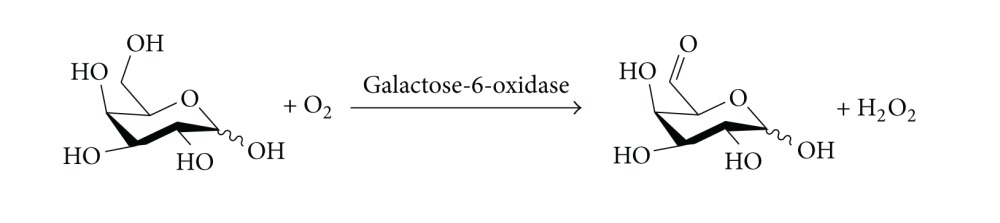
(3)


The enzyme is highly selective for C-6 of galactose, although it does tolerate substituents at the anomeric position of the galactose (i.e., the formation of glycosides). Possible galactose-derived by-products include the uronic acid (from overoxidation) or the *α*,*β*-unsaturated aldehyde (from E1_CB_ elimination across C-4–C-5). 

The oxidation of polysaccharides with galactose-6-oxidase has been investigated, but first, an optimisation of the reaction conditions was carried out on a monosaccharide model system, methyl *α*-d-galactopyranoside [133]. The best results were obtained using a combination of three enzymes (*viz* galactose-6-oxidase, catalase, and horseradish peroxidase) in water rather than buffer. Catalase (EC 1.11.1.6) was added to catalyse the decomposition of the H_2_O_2_ formed in the reaction, as otherwise H_2_O_2_ can poison the activity of the galactose-6-oxidase. Horseradish peroxidase was added to activate the oxidase enzyme by oxidising it to its active form.

**Scheme 10:**
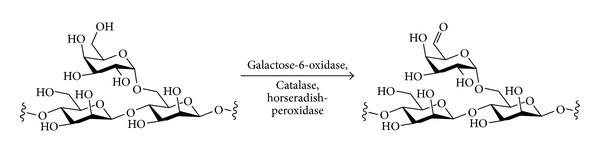
Enzymatic oxidation of guar gum.

The same group went on to investigate the oxidation of polysaccharides using galactose-6-oxidase in some detail [134]. The general oxidation procedure was as follows: the polysaccharide was stirred in water at 4°C or RT for 1–12 h until it had dissolved. Then the enzymes were added, and the mixture was stirred for 48 h. The oxidation of several galactose-containing polysaccharides was investigated using the same three-enzyme system. These included spruce galactoglucomannan [a *β*(1–4)-linked backbone of glucose and mannose residues with pendant galactose residues linked *α*(1–6)], guar gum [a *β*(1–4)-mannan backbone with pendant galactose residues linked *α*(1–6)], larch arabinogalactan [a *β*(1–3)-linked galactan backbone with pendant arabinofuranose units linked *α*(1–6), and galactose and galactobiose units, linked to the backbone by *β*(1–6)-linkages], corn arabinoxylan [a *β*(1–4)-linked xylan with various appendages, mostly arabinofuranose], and xyloglucan from tamarind seeds [a *β*(1–4)-linked glucan with pendant *α*(1–6)-linked xylose units; about half of the xylose residues are galactosylated]. Hence the polysaccharides had different galactose contents and different presentations of the galactose units due to branching, and the efficiency of the oxidation reaction varied between the different polysaccharides. Xyloglucan was the most efficiently oxidised (up to DS_ox_ 0.8 based on the galactose residues), followed by galactoglucomannan (DS_ox_ *ca* 0.65) and guar gum (DS_ox_ *ca* 0.4; Scheme 10).

There are also some further earlier reports on the oxidation of polysaccharides by galactose-6-oxidase in the literature. The galactose residues in guar gum were converted into the corresponding uronic acids in a two-step process consisting of enzymatic oxidation at C-6 with galactose-6-oxidase, followed by chemical oxidation (with I_2_/KI) [135]. A synthetic polysaccharide consisting of chitosan to which lactose had been attached by reductive amination was also a substrate for galactose-6-oxidase, and the appended galactose units could be oxidised enzymatically at C-6 [136]. The (1-deoxy-lactit-1-yl) chitosan was dispersed in phosphate buffer to give a soft glassy gel, which was purged with O_2_ for 1 min. Catalase and galactose-6-oxidase solutions were added and a viscous material formed after a few hours. After 2 d, the mixture was diluted with water, and the polysaccharide was precipitated from absolute ethanol to give a product with a DS_ox_ of *ca* 0.7.

### 4.3. Oxidative Cleavage of 1,2-Diols

Periodate may be used as an oxidising agent to achieve the ring-opening cleavage of the 1,2-diols at C-2 and C-3 of polysaccharides very efficiently and selectively. The initial product is the dialdehyde and is then usually oxidised further to give the dicarboxylate.

The C-2–C-3 oxidation mode was tested on starch and maltodextrin using different oxidants [121]. Tungstate/H_2_O_2_ and hypochlorite both resulted in chain degradation. The best results were obtained using a two-step procedure of periodate oxidation-cleavage (to the dialdehyde) followed by chlorite oxidation (to the dicarboxylate). Under the same conditions, the polysaccharides tested were essentially quantitatively ring-opened to give the polycarboxylate derivatives. It was also confirmed that (as expected) the ring-opened polymers are more susceptible than the parent unoxidised polysaccharides to acid-catalysed depolymerisation (i.e., acetal hydrolysis).

**Scheme 11:**
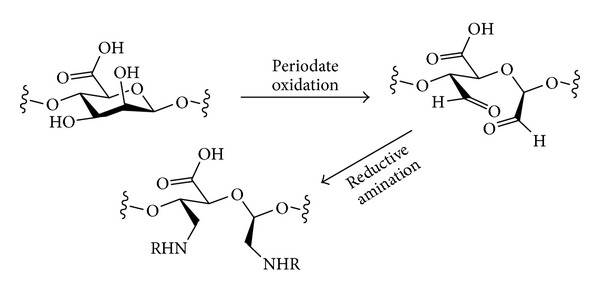


For cellulose, the efficiency of this oxidation reaction may be improved by the addition of metal salts to disrupt intermolecular hydrogen bonding and improve the solubility [137]. Alginates have been subjected to C-2–C-3 oxidative cleavage using periodate [138]. Initially formed aldehyde products were subjected to reductive amination with long-chain alkylamines to give hydrophobically modified derivatives (Scheme 11).

## 5. Reactions of Carboxylic Acids

**Scheme 12:**
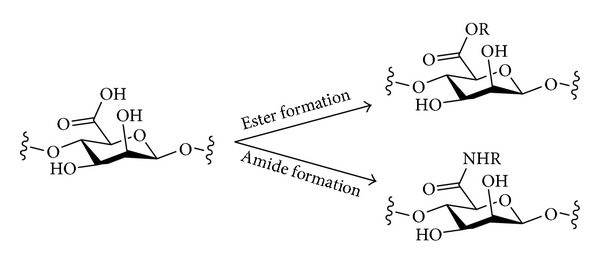


Several natural polysaccharides, including alginates and pectins, use uronic acid residues as structural components. In a uronic acid derivative, the C-6 position is oxidised to the carboxylic acid level. This section covers the reactions of these carboxylic acids (Scheme 12), both electrophilic and nucleophilic reactions, including esterification, amide formation, and multicomponent reactions. As well as natural uronic-acid-containing polysaccharides, this chemistry may be applicable to synthetic C-6 oxidised polysaccharides (see above). The modification of the carboxylic acid (uronic acid) functionality of alginates has been reviewed [138, 139].

### 5.1. Esterification

Carboxylic acids can react either as electrophiles or nucleophiles to form esters. In the first scenario, the acid must first be activated, which may happen prior to the esterification (e.g., by formation of an acid chloride), or *in situ* by using a coupling reagent such as DCCI, or by using a strong-acid catalyst (Fischer esterification). The activated acid should then be attacked by an alcohol nucleophile to give the ester. However, this approach has some disadvantages that mean it does not appear to have been widely used for the modification of polysaccharide uronic acids: (i) in aqueous solution, the water can effectively compete with the intended alcohol nucleophile, hydrolysing the activated acid intermediates and restoring the carboxylic acid starting material; (ii) where the other hydroxyl groups of the polysaccharide are unprotected, they too could compete as nucleophiles with the added alcohol, and possible cyclised products could result; (iii) in a Fischer (acid-catalysed) esterification, there is significant risk of depolymerisation of a polysaccharide substrate. 

In the second approach, the carboxylic acid can be deprotonated by a weak base to generate a carboxylate. This can then react as a nucleophile with alkylating agents to generate the esters. The hydroxyl groups of the polysaccharide will not normally react under these conditions, and so this approach has been more widely used for the preparation of esters of polysaccharide uronates [140, 141].

Treatment of the TBA salt of (completely demethylated) pectin with benzyl bromide and TBAI in DMSO at RT gave the benzyl ester with a DS of up to 0.73 [141]. The decyl ester could be prepared similarly with a DS of up to 0.44. The same method has been used for the preparation of esters of pectin with lower DS (>0.1) [142] and of alginates and hyaluronates, again with lower DS (>0.1) [140].

### 5.2. Amide Formation

Uronic acids must be activated to react as electrophiles with amine nucleophiles to generate amides. Classically, this can be achieved using a coupling (dehydrating) agent such as DCCI or the water-soluble EDCI, but even esters can be used as electrophilic carboxylic acid derivatives in amide-forming reactions.

The conversion of the uronic acids of alginate into amides has been achieved by reaction with amines in water using EDCI, a water-soluble coupling agent [139]. Alginate amides with DS of 0.1–0.3 were synthesised in this way by the reaction of sodium alginate with octylamine and EDCI in water [143]. Purification was achieved by precipitation from EtOH. Alternatively, the reactions could be carried out in an organic solvent. Thus, alginate amides with DS of up to 0.2 were prepared by the reaction of an alginate TBA salt with decylamine and CMPI (2-chloro-1-methylpyridinium iodide; the coupling agent) in DMF [144]. Purification was achieved by ion exchange followed by precipitation from water.

Esters react directly with amines to form amides in a reaction termed aminolysis. In a polysaccharide context, highly methylated pectin (methyl esters; DS_methyl_ = 0.73) was treated with various alkylamines (*n*-butyl up to *n*-octadecyl) in DMF under heterogeneous conditions (8, 25, or 45°C), and the amide products were formed with DS_amide_ = 0.4–0.55 [145–147].

### 5.3. Other Reactions

Other reactions of carboxylic acids may also be applicable to polysaccharide uronic acids. A concept that has been used to rapidly generate molecular diversity is that of multicomponent reactions [148–151], in which condensation/addition products are generated from three or more starting materials in a single reaction. Carboxylic acids are often found as components in such reactions.

**Scheme 13:**
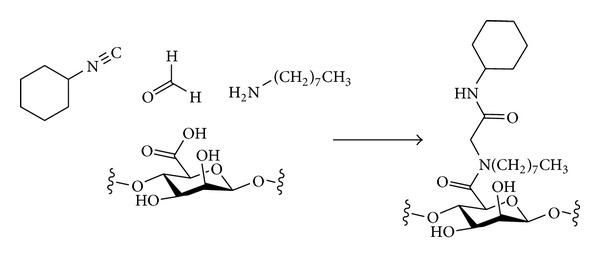
Ugi reaction of a polysaccharide.

One example is the Ugi four-component reaction between an aldehyde (or ketone), an amine, an isocyanide, and a carboxylic acid to form a diamide [152, 153]. It has been shown that the uronic acids of alginate can undergo the Ugi reaction (Scheme 13) [154]. Thus, an aqueous solution of alginate was treated with formaldehyde, octylamine, and cyclohexyl isocyanide for 24 h. Purification was achieved by dialysis.

## 6. Saccharide Nitrogen as Nucleophile

This section concerns the reactions of polysaccharide amines such as chitosan, which carries a free basic nitrogen at C-2, but the methods should also be applicable to other synthetic aminated polysaccharides, for example, C-6 aminated cellulose.

Amines can react with electrophiles to give amides (i.e., acylation), higher order amines or ammonium salts (i.e., alkylation), or imines (Schiff bases). The different reactivity of nitrogen and oxygen nucleophiles means that it is often possible to carry out these derivatisations in aqueous solution, and without protection of any free hydroxyl groups in the saccharide derivative. Of course, *O*-alkylation and *O*-acylation may take place under some conditions, but with an appropriate choice, it should be possible to find conditions that favour chemoselective derivatisation at nitrogen. 
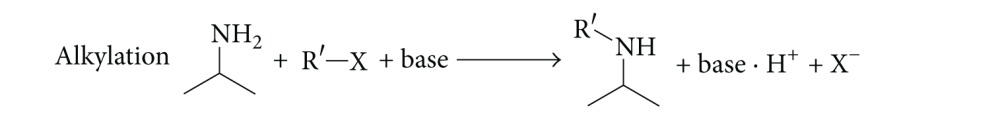
(4)




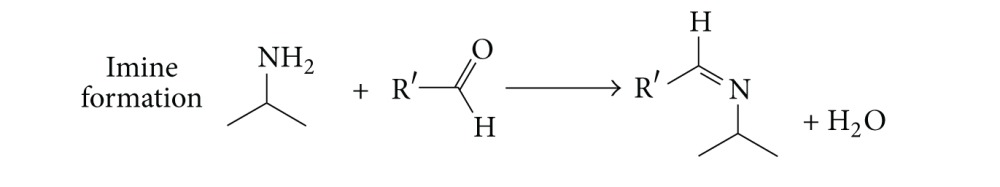
(5)




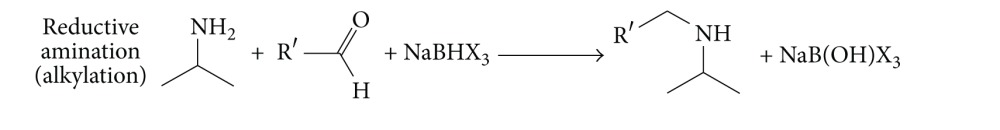
(6)




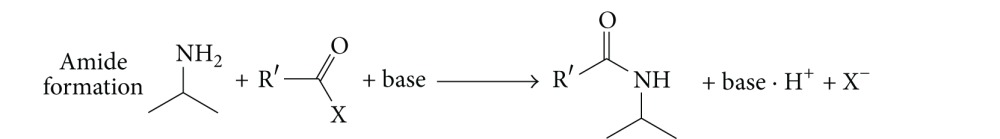
(7)


The alkylation of amines can be complex in that the initial products, which are also amines, can react further to form higher order amines or, under direct alkylation conditions, eventually ammonium salts. This can be particularly problematic in direct alkylation reactions with very reactive electrophiles (sterically, e.g., methyl; electronically, e.g., benzyl; or with special reactivity, e.g., allyl) and with reactive nucleophiles. As a result, direct alkylation is not normally used for the preparation of amines, even though when the reactants are more sterically hindered, as is the case with saccharide amine nucleophiles and moderately hindered electrophiles, the barrier to oversubstitution increases.

The reductive amination reaction is widely regarded as the alkylation method of choice for amines. In this method, the amine first condenses with a carbonyl compound (normally an aldehyde) to give an imine. A reducing agent, normally NaBH_4_, NaCNBH_3_, or Na(OAc)_3_BH, reduces the imine to give the amine product. The reaction is best carried out under mildly acidic conditions. Overalkylation can be minimised by this method, but in fact, it is still often seen to a greater or lesser extent (see below). But quaternisation to form ammonium salts cannot occur under these conditions and neither can *O*-alkylation to form ethers, and these are definite advantages over a direct alkylation method.

### 6.1. Reductive Amination

**Scheme 14:**
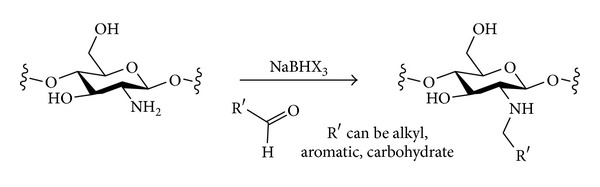


A standard procedure for the preparation of *N*-alkylated derivatives of chitosan by reductive amination has been widely used over the years (Scheme 14) [136].

Even here, though, overalkylation occurs and products with homogeneous structures are often not obtained. Depending on the ratio of GlcN/aldehyde used, the polysaccharide products were composed of mixtures of mainly monoalkylated and unalkylated glucosamines or mainly dialkylated and monoalkylated glucosamines, according to the ^1^H NMR spectra of the products [155]. The general procedure is as follows: chitosan was dissolved (i.e., reactions are homogeneous) in either a mixture (1 : 1, pH 5.5) of an alcohol (normally methanol or ethanol) and 1% aq. acetic acid or in 1% aq. acetic acid alone. A solution containing the carbonyl compound and NaCNBH_3_ (7 equiv.) was added, and the reaction mixture was stirred at room temperature, usually until gel formation was observed (*ca *1–24 h). The reaction may be stopped by adjustment of the pH to 10. The solid product is then obtained by filtration and washing with methanol and Et_2_O. Further purification by Soxhlet extraction into EtOH/Et_2_O (1 : 1) has also been done in some cases [156, 157]. When no alcohol cosolvent is added, the reaction takes place in essentially aqueous solution. The role of the alcohol is to solubilise the aldehyde component, which can often be hydrophobic.

This procedure has been used with many different carbonyl components, including reducing monosaccharides, disaccharides, ketosugars, other oxidised sugars, and noncarbohydrate carbonyls [136]. Aldehydes bearing straight-chain alkyl groups with chain lengths from C_3_–C_12_ have been used [155]. Chitosan underwent *N*-alkylation under reductive amination conditions with benzylic (heterocyclic) aldehydes: furfural, methylfurfural, pyridine-3-carboxaldehyde, and so forth. The DS of the products was between 0.30 and 0.43, and the broad ^1^H NMR spectra showed two sets of signals, presumably due to the monoalkylated and the unalkylated glucosamines [156]. Chitosan underwent *N*-alkylation by reductive amination with aliphatic aldehydes C_2_–C_12_ (0.1 to 1 equiv.) to give products with DS between 0.03 and 0.3, and with twelve substituted benzaldehydes (1 equiv.) to give products with DS between 0.2 and 0.5 [157]. A fluorescence label was installed into chitosan by the reductive amination method with 9-anthraldehyde as the carbonyl component, aiming for very low DS (values between 0.00001 and 0.01) [158].

### 6.2. Imine Formation

**Scheme 15:**
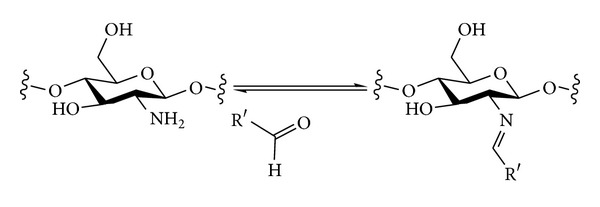


Imines, the C=N intermediates in the reductive amination procedure, are liable to hydrolyse—their formation is reversible. This is clearly a disadvantage when designing a stable product, but in cases where the reversible formation of semistable covalent compounds is beneficial, in supramolecular chemistry, for example, imines can be useful compounds. The conversion of chitosan into imines (without reduction; Scheme 15) has been investigated in solution (to give products with DS of *ca* 0.9) and under heterogeneous conditions on prespun polysaccharide fibres (to give products with DS of 0.9–1.0) [159]. Typical conditions for imine formation under homogeneous conditions are as follows: chitosan was dissolved in a mixture of 2% aq. AcOH and methanol, and a solution of the aldehyde in methanol was added. This mixture was left overnight, and then the imine (a solid/gel) was then purified by filtration and washing with methanol. Imine formation on prespun chitosan fibres was simply carried out by suspending the fibres in methanol and adding the aldehydes, and after the mixture had been left overnight, the derivatised fibres were washed with methanol.

### 6.3. Formation of Quaternary Ammonium Salts

**Scheme 16:**
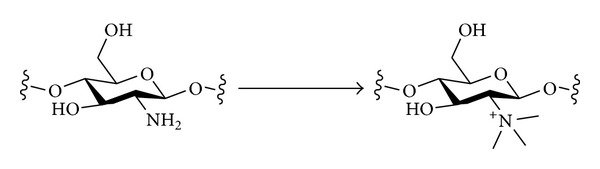


Repeated alkylation of the free amine base of chitosan eventually gives quaternary salts (Scheme 16). According to a very recent review covering the formation of quaternary salts (quaternisation) of chitosan [160], better synthetic routes that do not require the use of dangerous alkylating agents still need to be developed.

Much research into the quaternisation of chitosan has focussed on trimethyl derivatives [161]. In this transformation, the chitosan nitrogen must act as a nucleophile, attacking an alkylating agent (methylating agent) three times. The oxygen nucleophiles in chitosan (i.e., OH-3 and OH-6) could also be alkylated in a potential undesired side process. The pH of the reaction mixture can affect the rate and outcome of the reaction. When no base is added, the basic nitrogens in the starting material and partially alkylated products will be protonated, decreasing their nucleophilicity and resulting in products with low DS. But under basic conditions, *O*-alkylation could become problematic.

The methylation of chitosan with the aim of tri-*N*-methylation to form the quaternary ammonium salt without concomitant *O*-methylation has been investigated in some detail [162], and errors in a published method [163] were found. Thus, when alkylation was carried out with MeI and NaOH in 1-methyl-2-pyrrolidinone at 60°C, the major product was found to be the dialkylated product (i.e., the tertiary amine), and significant quaternisation did not occur. A polysaccharide with a DS_quat_ of 0.7 was obtained in a two-step procedure in which the initial product (containing the *N*,*N*-dialkylated material as its major component) was isolated and then resubjected to the same reaction conditions. But for higher DS_quat_ values, looking towards complete quaternisation, concomitant *O*-alkylation started to become significant.

A recent paper describes how a change of solvent can suppress *O*-methylation, enabling a one-pot synthesis of essentially uniform (DS *ca* 0.9) quaternised trimethyl chitosan [161]. In this approach, DMF/H_2_O (1 : 1) was used as solvent, and several separate additions of NaOH and MeI were necessary for complete quaternisation to be achieved. Purification of the products was achieved by precipitation, ion exchange, and dialysis.

A two-step approach to the synthesis of quaternised chitosan using reductive amination followed by alkylation opens the possibility of installing two different R groups onto the nitrogen atoms [164]. The reductive amination procedure was carried out essentially as described above. Subsequently, alkylation was carried out with MeI and NaOH in NMP as solvent, and purification was by precipitation from acetone. The chitosan derivatives obtained by this method were found electrochemically to have DS_quat_ values between 0.8 and 0.9.

### 6.4. Acylation (Amide Formation)

**Scheme 17:**
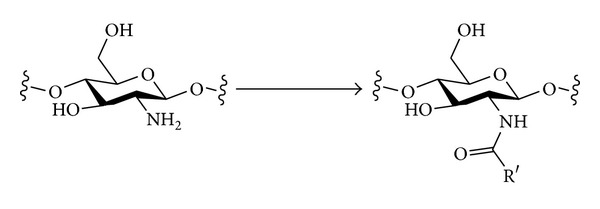


The acylation of amines to give amides (Scheme 17) is a very well investigated reaction, due to its importance in peptide synthesis. Here, I am covering the reaction of polysaccharide amines with nonpolysaccharide acylating agents to give amides [165, 166]; the related amide-forming reactions between polysaccharide carboxylic acid (uronic acid) derivatives and nonpolysaccharide amines following similar principles are covered above. The reaction may be carried out (in water or alcohol solvents) using acylating agents such as acyl chlorides or acid anhydrides, or using carboxylic acids and dehydrating agents. It can be beneficial to use a reactive *O*-nucleophile, such as water, methanol, or ethanol, as solvent or cosolvent so as to suppress *O*-acylation of the polysaccharide, a possible side-reaction that can occur when a polar aprotic solvent (such as DMF, NMP) is used.

Chitosan was *N*-acylated under homogeneous conditions in solution in 1% aq. AcOH and methanol (1 : 1) using different carboxylic anhydrides as acylating agents [167]. A solution of the anhydride in methanol was added to the chitosan solution, and the reaction was quenched after 15 min by pouring into ammonia solution (7 : 3, v/v). The precipitated polysaccharides were filtered and washed with methanol and ether. The DS values of the products were determined by titration to be <0.5.

Chitosan was also shown to undergo *N*-acylation under heterogeneous conditions. Fibres of the polysaccharide were suspended in methanol, and a carboxylic acid anhydride (5 equiv.; acetic, propionic, butyric, or hexanoic anhydride) was added. The mixture was shaken at 40°C for 24 h, and then the derivatised fibres were washed with methanol. The DS of the products were between 0.65 and 0.85, as determined by elemental analysis [168].

## 7. Unsaturated Derivatives

Polysaccharide derivatives in which the monosaccharide constituents contain C=C double bonds have been prepared. These C=C double bonds represent unusual types of functional groups in polysaccharides.

**Figure 4:**
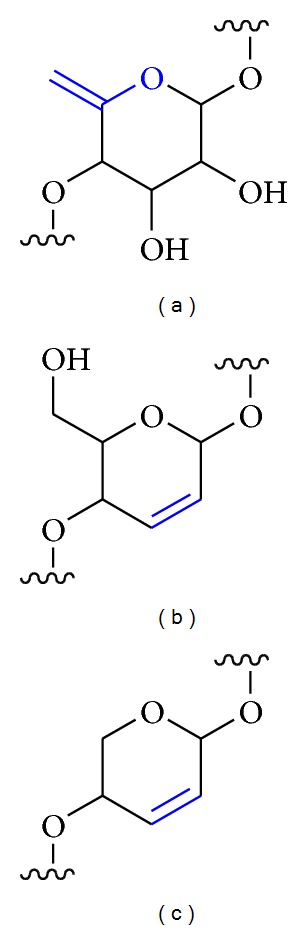
Unsaturated derivatives. (a) 5,6-Unsaturated (enol ether); (b) 2,3-unsaturated (alkene); (c) 2,3-unsaturated pentose derivative.

Cellulose derivatives of this type have been termed *cellulosenes *[5], and they should be classified as one of two types-enol ethers or alkenes—depending on whether one of the carbons of the C=C double bond is directly bonded to an oxygen or not (Figure 4). The enol ether and alkene types of unsaturated polysaccharides may be expected to have different properties and reactivities. 5,6-Cellulosene is unsaturated between C-5 and C-6 it is formed by simple elimination (i.e., a formal elimination of water from cellulose), and the C=C double bond is part of an enol ether. In 2,3-cellulosene, unsaturated between C-2 and C-3, the C=C double bond represents an alkene (olefin) functionality and must be formed by a reductive elimination from cellulose.

Some similar unsaturated derivatives of other polysaccharides have been synthesised. Xylan and amylose, two more common (1–4)-linked polysaccharides, have both been transformed into their 2,3-unsaturated olefinic derivatives. The 5,6-unsaturated (enol ether) derivative of amylose has also been investigated—of course, as xylose is built up of pentose monomers, a corresponding 5,6-unsaturated derivative of this polysaccharide cannot exist.

Further possibilities for both the enol ether and alkene types of unsaturated polysaccharide can be envisaged. For (1–6)-linked structures, olefinic unsaturation in the ring could be located either between C-2 and C-3 or between C-3 and C-4, although the regioselective synthesis of such compounds may not be straightforward. In (1–3)-linked pyranose-based polysaccharides, an alkene structure is impossible, as all of C-1, C-3, and C-5 must bear an oxygen atom. (1–2)-Linked pyranose-based polysaccharides are not common.

For both simple elimination and reductive elimination reactions, stereoelectronic factors are important. It will normally be necessary for the two groups that will undergo the elimination reaction to adopt an antiperiplanar or synperiplanar relationship. Free rotation about the exocyclic C-5–C-6 bond should allow a favourable conformation to be reached in the synthesis of 5,6-unsaturated polysaccharides. For the synthesis of compounds with endocyclic unsaturation, though, the stereochemistry of the hydroxyl groups in the pyranose ring can be important.

### 7.1. 5,6-Unsaturated Derivatives

As stated above, the formal overall process for the synthesis of an enol-ether-based unsaturated derivative of a polysaccharide is elimination of water. For 5,6-unsaturated derivatives this means elimination of water across C-5 and C-6. In a two-step process, OH-6 is converted into a good leaving group, and then treatment with a base will promote the elimination reaction. Processes for the regioselective conversion of OH-6 into a good leaving group are quite well described (see the section on nucleophilic substitution, above). It is well known that nucleophilic substitution reactions can compete with basic eliminations. Such competing processes are typically minimised by using a nonnucleophilic (e.g., sterically hindered) base. However, in polysaccharide systems, when the polysaccharide is unprotected, any base could deprotonate the free hydroxyl groups in the pyranose rings to generate intramolecular nucleophiles that could attack the carbon bearing the leaving group to form a new ring. The undesired intramolecular cyclisation of O-3 onto C-6 in particular has been a problem in the synthesis of 5,6-cellulosene.

**Scheme 18:**
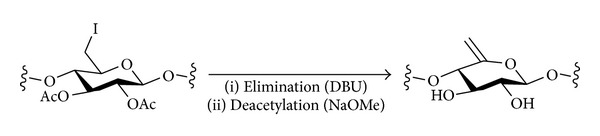


A solution to this problem has been reported in a synthesis of 5,6-cellulosene that gave a DS as high as 0.7 (Scheme 18). HI was eliminated from 2,3-di-*O*-acetyl-6-deoxy-6-iodocellulose by treatment with DBU [169]. DBU is a strong, nonnucleophilic base that is able to induce elimination without acting as a nucleophile on C-6 or removing the acetate protection from O-2 or O-3. The acetates were subsequently cleaved by methoxide treatment to give the unprotected polysaccharide derivative.

### 7.2. 2,3-Unsaturated Derivatives

2,3-Unsaturated derivatives of the (1–4)-linked polysaccharides cellulose [5], amylose [170], and xylan [170] have all been mentioned in the literature. The stereochemistry at C-2 and C-3 of all these polysaccharides is the same, i.e., *trans* diequatorial, which means that they may be expected to form 2,3-unsaturated polysaccharides under similar conditions (Scheme 19).

The conversion of amylose into its 2,3-unsaturated derivative was achieved by the following reaction sequence [170]: protection of O-6 as a trityl ether; conversion of O-2 and O-3 into tosylates; reductive elimination with zinc and sodium iodide. Xylan was converted into the corresponding unsaturated polysaccharide following a similar sequence. The reactivity of the alkene functionality was also briefly investigated, undergoing dibromination or hydrogenation [170].

The number of published methods for the synthesis of alkene-containing polysaccharides by reductive elimination is limited, but studies of similar reactions on simpler monosaccharide systems can be relevant for the further development of this chemistry. A one-step procedure [171] to convert pyranoside 2,3-diols into alkenes seems particularly relevant. Treatment of the diols with chlorodiphenylphosphine, iodine, and imidazole (reflux, 1 h) gave 2,3-unsaturated derivatives in 75–89% yields starting from glucose (2,3-*trans*) derivatives, and in 52% yield from a mannose (2,3-*cis*) derivative. Alternatively, *vic*-diols were first converted into *vic*-halocarboxylates, which were then treated with a reducing agent such as zinc [172, 173] or NaSH [174] to give the alkenes. The reductive elimination step can be easier for furanoside than pyranoside substrates [174].

## 8. Concluding Remarks

As well as summarising the achievements in this field, also the gaps are highlighted, and this will hopefully inspire further developments. Many of the methods that have been developed for the modification of polysaccharides are inefficient and wasteful, as stoichiometric amounts of waste products may be formed and several steps may be required. The use of a renewable resource loses a lot of its meaning and significance if it must undergo many manipulations with nonrenewable materials before reaching its final goal. Thus, future research in this area would do well to focus on catalytic transformations.

## Figures and Tables

**Scheme 19:**